# Fgf10/Fgfr2b Signaling Orchestrates the Symphony of Molecular, Cellular, and Physical Processes Required for Harmonious Airway Branching Morphogenesis

**DOI:** 10.3389/fcell.2020.620667

**Published:** 2021-01-12

**Authors:** Matthew R. Jones, Lei Chong, Saverio Bellusci

**Affiliations:** ^1^Key Laboratory of Interventional Pulmonology of Zhejiang Province, Department of Pulmonary and Critical Care Medicine, The First Affiliated Hospital of Wenzhou Medical University, Wenzhou, China; ^2^Cardio-Pulmonary Institute and Department of Pulmonary and Critical Care Medicine and Infectious Diseases, Universities of Giessen and Marburg Lung Center (UGMLC), Member of the German Center for Lung Research (DZL), Justus-Liebig University Giessen, Giessen, Germany; ^3^National Key Clinical Specialty of Pediatric Respiratory Medicine, Discipline of Pediatric Respiratory Medicine, Institute of Pediatrics, The Second Affiliated Hospital of Wenzhou Medical University, Wenzhou, China

**Keywords:** Fgfr2b, Fgf10, branching morphogenesis, lung, epithelial-mesenchymal cross-talk

## Abstract

Airway branching morphogenesis depends on the intricate orchestration of numerous biological and physical factors connected across different spatial scales. One of the key regulatory pathways controlling airway branching is fibroblast growth factor 10 (Fgf10) signaling *via* its epithelial fibroblast growth factor receptor 2b (Fgfr2b). Fine reviews have been published on the molecular mechanisms, in general, involved in branching morphogenesis, including those mechanisms, in particular, connected to Fgf10/Fgfr2b signaling. However, a comprehensive review looking at all the major biological and physical factors involved in branching, at the different scales at which branching operates, and the known role of Fgf10/Fgfr2b therein, is missing. In the current review, we attempt to summarize the existing literature on airway branching morphogenesis by taking a broad approach. We focus on the biophysical and mechanical forces directly shaping epithelial bud initiation, branch elongation, and branch tip bifurcation. We then shift focus to more passive means by which branching proceeds, *via* extracellular matrix remodeling and the influence of the other pulmonary arborized networks: the vasculature and nerves. We end the review by briefly discussing work in computational modeling of airway branching. Throughout, we emphasize the known or speculative effects of Fgfr2b signaling at each point of discussion. It is our aim to promote an understanding of branching morphogenesis that captures the multi-scalar biological and physical nature of the phenomenon, and the interdisciplinary approach to its study.

## Introduction

A prerequisite for life is the exchange of material and information between the internal and external milieu. An organism must balance the intake, distribution, and expulsion of the resources necessary for life, not only between itself and the outside environment, but also within and among the cells and organs of its body. During the transition from single-celled organisms to multicellular plants and animals, the demand for efficient and robust exchange of resources has scaled accordingly. A common solution to this demand has been to increase the surface area of the cells or tissues facilitating the exchange with the environment. Since the given volume available to any network of cells or tissues is limited, organisms have consistently evolved cellular and tissue networks of higher-order structural geometries to efficiently and optimally increase the surface area to volume ratio. The physical process by which these higher-order structures develop is called branching morphogenesis, whereby cells or tissues undergo branch specification, followed by iterations of elongation and branching to form an arborized scaffold supporting the functional units of resource exchange.

Branching structures are found throughout nature. An obvious example is plants, many of which are essentially branched vascular networks supporting the leaves which represent the functional units of energy and gas exchange. In mammals, branching morphogenesis accounts for the multiple dendrites of a single nerve cell, the complex vascular network responsible for blood transportation and gas exchange, and the many branched organs including the mammary, salivary, and lacrimal glands as ectoderm-derived organs, as well as the kidney, pancreas, and the lung as endoderm-derived organs. The result of branching morphogenesis in each of these cases is the optimal space-filling occupancy of functional units of exchange (see reviews by Ochoa-Espinosa and Affolter, 2012; Spurlin and Nelson, 2017).

Remarkably, for such a seemingly complex process, researchers have identified and continue to investigate a relatively limited set of principles and molecular and biophysical mechanisms by which branching morphogenesis proceeds. This is a consequence of evolutionarily conserved molecular control mechanisms, such as ancient signaling pathways, which function, albeit with species and organ specific modifications, in a similar fashion throughout the living world. There are, in addition, purely physical means by which a structure can branch, which depend upon a set of rules that can be computationally modeled. Researchers are beginning to wed these physical rules to our biological understanding of branching morphogenesis, painting an intriguing picture of the regulatory interplay between physics and biology (see reviews by Iber and Menshykau, 2013; Lang et al., 2018).

The mammalian lung is a prime example of an organ which forms via branching morphogenesis. For example, the adult human lung is comprised of around 17 million airway branches supporting in the range of half a billion alveoli, and arterial and venous trees which are composed of over 250,000 arterioles and a delicate network of capillaries surrounding each alveolus. This entire structure has a surface area estimated in the range of 70–130 m^2^, yet occupies a volume of roughly 5–6 l, which is equivalent to packing a standard piece of paper into a thimble (reviewed in Glenny, 2011).

Understanding the principles and mechanisms regulating branching morphogenesis in the mammalian lung continues to be an active area of research. A number of critical molecular pathways and biophysical properties have already been elucidated. What emerges from this research is an understanding that a relatively small and conserved set of principles and mechanisms can, when appreciated together, explain the process of branching morphogenesis in the lung. In other words, branching morphogenesis depends on an intricate orchestration of multiple factors, including signaling pathways facilitating epithelial-mesenchymal crosstalk; cellular and tissue rearrangements and shape changes; and purely physical and mechanical forces.

Airway branch development can be divided into different stages, defined by distinct morphologies and cellular and molecular regulatory mechanisms. These include monopodial branch initiation, where the lateral epithelium of an existing airway bulges outward forming a branch bud; directed branch elongation, where the newly formed bud extends into the surrounding mesenchyme in a coordinated manner; and branch arrest and tip bifurcation, where the branch ceases to elongate, and the distal tip dilates and assumes a characteristic flattened phenotype before undergoing planar or orthogonal bifurcation (or, far less commonly, trifurcation).

In this review, after a general overview of lung branching morphogenesis in the mouse, we will discuss each of these three stages in the context of Fgfr2b signaling. Fgfr2b signaling is a master conductor of lung branching, and plays a role in most of the principles controlling branching in the lung. We will emphasize not only the molecular and cellular regulation of morphogenesis at each stage, but also, when possible, biomechanical and biophysical considerations. Branching morphogenesis is a result of interconnected biological and physical mechanisms operating at multiple spatial and temporal scales. Therefore, our aim in this review is to comprehensively integrate these mechanisms into a unified picture, thereby gaining a broad and robust overview of Fgfr2b signaling orchestration of lung branching morphogenesis.

## Overview of Mouse Lung Branching Morphogenesis

**Figure 1:**
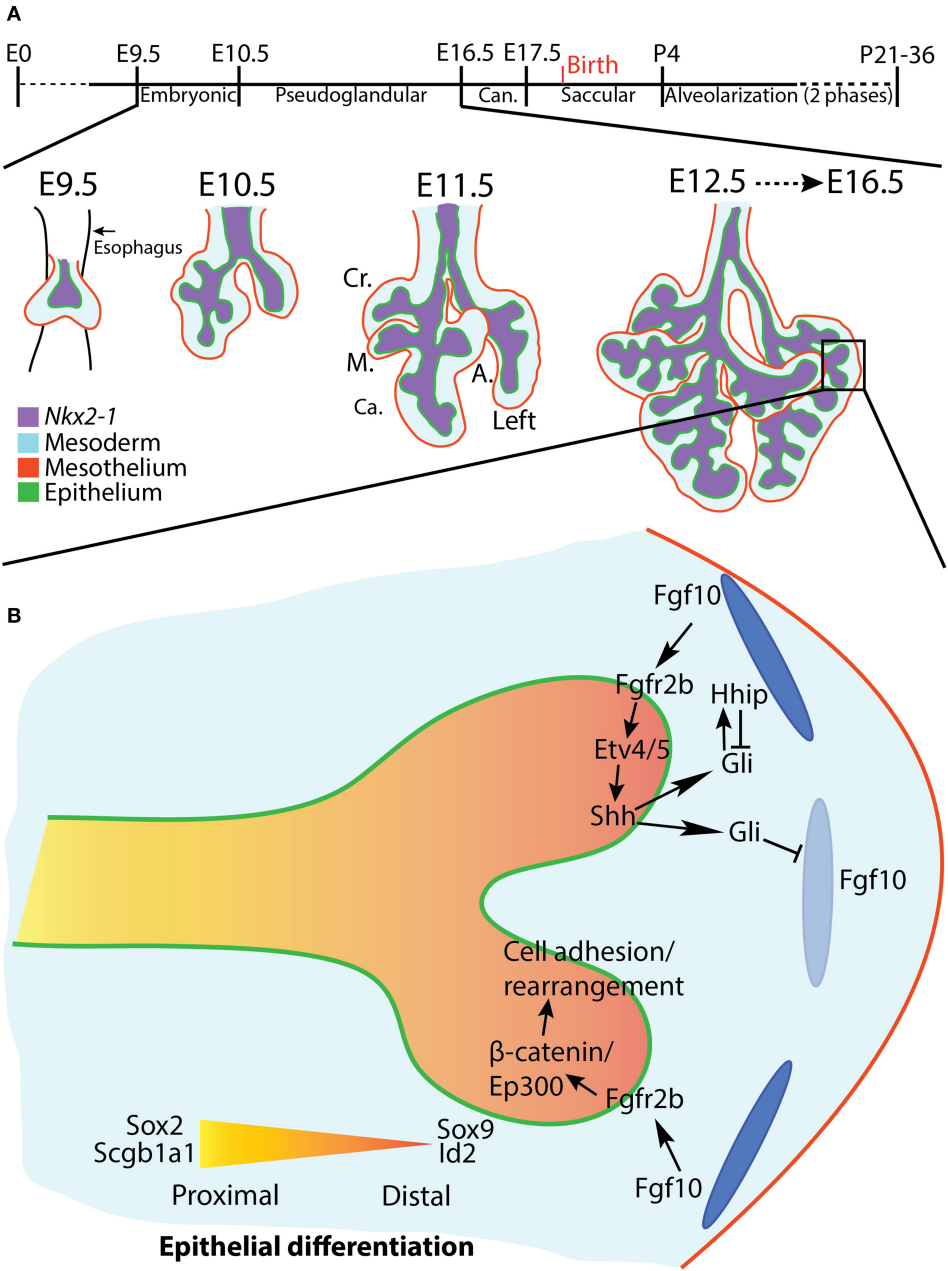
Overview of mouse lung development and early pseudoglandular branching morphogenesis. **(A)** Stages of mouse lung development (see text for details). A lung in earliest stages of branching morphogenesis is depicted. The four right lobes and left lobe are discernable at E11.5. Cr., Cranial; M., Medial; Ca., Caudal; A., Accessory. **(B)** A distal lung bud at E12.5, shortly after bifurcation. The top branch highlights the Fgf10/Fgfr2b/Etv/Shh regulatory pathway. The bottom branch depicts regulation of cell adhesion and rearrangement *via* the Fgf10/Fgfr2b/β-catenin/Ep300 pathway. Distal airways are marked by Sox9 and Id2 expression, whereas proximal airways express Sox2 and Scgb1a1. Dark and light blue ovals represent localized sources of high and low Fgf10 expression, respectively.

Lung development in the mouse begins around embryonic day (E) 9.5 as the anlage of the future trachea and lung evaginate from a specialized region of *Nkx2-1* expression on the anterior foregut endoderm. The development of the lung proceeds through a series of morphologically-distinct stages, beginning with the embryonic stage (E9.5-E10.5) (Taghizadeh et al., 2020) and proceeding through the pseudoglandular stage (E10.5-E16.5), canalicular stage (E16.5-E17.5), saccular stage (E17.5-postnatal day (PN) 4), and ending after two phases of an alveolarization stage (P4-young adulthood) (reviewed in Schittny, 2017) (Figure 1A).

After the two primary bronchi of the mouse lung form at E9.5, they elongate and grow into the surrounding mesenchyme. This initial bronchial formation and growth is *not* considered the first branch of the future bronchial tree. Branching morphogenesis *per se* begins around E10.5, as the primary bronchi begin ordered iterations of bud initiation, elongation, and, for dichotomous branches, bifurcation. Branch epithelium can be divided based on geographical, cellular, and molecular criteria into proximal (the stalk) and distal (the tip) regions. The proximal epithelium is composed of relatively differentiated cells, and is often demarcated histologically by the expression of Sox2 and Scgb1a1; the distal epithelium contains airway progenitor cells characterized by Sox9 and Id2 expression (Figure 1B). Branching morphogenesis occurs primarily during the pseudoglandular (E10.5-E16.5) and canalicular (E16.5-E17.5) stages of development, and ends after 13–17 branch generations to produce the bronchial tree of conducting airways, each of which terminates distally in acinar buds which will eventually form the alveoli (for a review see Cardoso and Lü, 2006).

Following a seminal paper by Metzger et al. (2008), it was hypothesized that mouse lung branching morphogenesis is controlled by genetically encoded subroutines, which operate reiteratively during morphogenesis. In this paper, the authors demonstrated *via* detailed *in vivo* mapping of branching over embryonic development that lung epithelium branches occur in a precise and predictable spatio-temporal order relative to the other branches on the tree. These authors identified three modes of branching: monopodial domain branching, where buds branch from the periphery of existing branches; and dichotomous planar bifurcation and orthogonal bifurcation, which are distinguished according to their orientation relative to the parent branch. The idea of genetically encoded subroutines controlling morphogenesis is still an area of research, but it may prove to be false, especially after the first generation of branching, when both spatial and temporal variations become common. Instead, it has been suggested that ordered branching can be explained by coupled interactions between airway epithelium and the surrounding mesenchyme, which results in the space-filling expansion of the airways, the geometry of which is defined locally by the shape of the mesoderm, and ultimately by the shape of the thoracic cavity (Blanc et al., 2012; Clément et al., 2012). While research continues on the causal mechanisms regulating branching morphogenesis, the basic stereotypy of lung branching, and the nomenclature employed to describe it, are widely accepted in the literature.

A number of evolutionarily conserved signaling pathways have been implicated in lung domain specification, lung bud evagination, and subsequent branching morphogenesis. These pathways include bone morphogenic protein (Bmp), epidermal growth factor (Egf), sonic hedgehog (Shh), transforming growth factor beta (Tgf-β), vascular endothelial growth factor (Vegf), Wnt, and fibroblast growth factor (Fgf) signaling. Each of these pathways is involved in the intricate and critical epithelial-mesodermal crosstalk necessary to initiate and coordinate branching morphogenesis. This crosstalk typically involves a diffusible ligand from the surrounding mesoderm signaling to the adjacent epithelium through a cognate receptor. Upon activation of downstream signaling and regulation of target genes and proteins, other ligands are secreted from the epithelium to activate or repress targets in the mesenchyme. Such crosstalk often produces negative feedback loops, which are necessary to coordinate axis and branching patterns during development, to ensure proper spatio-temporal expression of morphogens in the mesenchyme, and to maintain a distal tip progenitor and organizing center (for reviews see Warburton et al., 2005; Morrisey and Hogan, 2010; Swarr and Morrisey, 2015; Zepp and Morrisey, 2019).

A powerful example of epithelial-mesodermal crosstalk is the Fgf10-Etv4/5-Shh feedback loop. In this case, Fgf10 is expressed from the sub-mesothelial mesenchyme around the distal epithelial buds (Bellusci et al., 1997). Fgf10 signals through its epithelial receptor fibroblast growth factor receptor 2b (Fgfr2b) and upregulates the expression of *Etv4* and *Etv5* (Herriges et al., 2015; Jones et al., 2019b). These transcription factors in turn upregulate the expression of *Shh*. Shh is secreted from the epithelium and, depending on its concentration, either represses *Fgf10* expression or upregulates its own antagonist, hedgehog interacting protein (*Hhip*), whereupon levels of Fgf10 are maintained. In this manner, localized expression of Fgf10 in the mesenchyme dynamically shifts over time, remaining concentrated around the distal bud tips, and is downregulated in the bud clefts during bifurcation, and in the proximal stalks, where levels of Hhip are lower. This dynamic feedback mechanism also interacts with many of the other pathways and feedback loops controlling branching morphogenesis (for example, Shh also interacts with Bmps and Wnts) in an intricate regulatory web (reviewed in Swarr and Morrisey, 2015).

A recent paper published by our lab identified a comprehensive set of direct epithelial transcriptional targets of Fgfr2b signaling, the “Fgf10 gene signature,” in E12.5 mouse lungs (Jones et al., 2019a). Not surprisingly, it was found that these genes control a swathe of biological processes, the most significant of which at this stage being cell-cell and cell-matrix adhesion. Furthermore, it was suggested that a majority of the “Fgf10 signature” is regulated *via* β-catenin/Ep300 transcriptional activity acting downstream of Fgfr2b activation, thus potentially revealing yet another critical signaling axis controlling branching morphogenesis (Figure 1B).

### Mechanisms of Branching Morphogenesis in the Context of Fgfr2b Signaling

Morphogenesis is, by definition, a physical process; it is the movement and rearrangement of individual cells and tissues from one geometric configuration to another. Each cell must respond to extracellular information to properly coordinate its movements, and in some sense must “know” its spatio-temporal location relative to other cells in the developing lung. The information guiding a cell's decisions is encoded by extracellular signaling molecules, such as morphogens, as well as by biomechanical and other physical forces. While early research focused primarily on the molecules involved in branching morphogenesis, there is increasing attention on the mechanical regulations of branching, and the interconnection between physics and biology at multiple scales. This attempt to understand the interconnected mechanisms regulating different branching structures, and to uncover potential unifying principles across structures and species, has led to exciting research not only in molecular biology, but also in biomechanics and engineering, computational biology, and mathematical modeling.

Molecular control of branching morphogenesis depends on an interplay of inductive stimuli and counteracting inhibitors (Horowitz and Simons, 2008), and the molecules underlying branching morphogenesis are remarkably conserved among organs and phyla (reviewed in Nelson and Gleghorn, 2012). That being said, however, there is a wide range of mechanisms by which the conserved molecules effect morphological change, both within similar structures among different species and among different structures within the same species (Varner and Nelson, 2014).

Spurlin and Nelson (2017) propose three general mechanisms by which branched networks form: single cell extension (e.g., nerves); collective migration (e.g., blood vessels, mammary glands); and non-migratory branching (e.g., kidneys, lungs). The multicellular tissues of branched organs form either through directed collective migration or through mechanisms independent of active migration. Branches forming through collective migration contain cells at their tips called “leader cells.” These cells extend filopodia into the surrounding mesenchyme, producing traction. By maintaining tight connections with the trailing cells of the stalk, and by increasing cell proliferation at the tip, leader cells actively invade surrounding mesenchyme to establish new branches.

Initially, it was believed that lung epithelium branched according to collective migration principles. It was discovered that nascent buds formed adjacent to localized mesenchymal expression of Fgf10 (Bellusci et al., 1997), which was hypothesized to initiate bud formation, perhaps through an increase in localized proliferation of epithelial cells, and serve as chemotaxic signaling centers, guiding actively migrating cells. In *in vitro* studies, wherein recombinant FGF10 soaked beads were placed adjacent to mesenchyme-free epithelium isolated from developing lungs and cultured 3-dimensionally in reconstituted basement membrane (Matrigel), the epithelium branched and grew toward the localized source of FGF10 expression (Park et al., 1998; Lebeche et al., 1999; Weaver et al., 2000; Lü et al., 2005). In other 3D culture experiments, however, where rFGF10 was added ubiquitously to the Matrigel, and therefore any localized source of FGF10 was absent, the embedded epithelium still underwent branching (Bellusci et al., 1997; Park et al., 1998; Ohtsuka et al., 2001). Furthermore, in *in vivo* studies using *Fgf10*^−/−^ knockout mice which were crossed with mice allowing the conditional and ubiquitous expression of Fgf10 in the mesenchyme, it was demonstrated that branching proceeded relatively normally upon ubiquitous Fgf10 expression (Volckaert et al., 2013). These studies indicate that additional investigation is required to settle the precise role played by Fgf10 during branching.

The fact remains, however, that Fgf10 signaling through its cognate receptor is necessary for branching morphogenesis. Constitutive loss of either *Fgf10* or *Fgfr2b* leads to complete lung agenesis apart from the initial bronchi (Sekine et al., 1999; De Moerlooze et al., 2000), whereas conditional inhibition of Fgf10/Fgfr2b signaling produces more or less drastic impairment of branching morphogenesis, depending on the time-point of inhibition (Jones et al., 2019a, 2020; Taghizadeh et al., 2020). Given that the initial hypothesis of localized Fgf10/Fgfr2b signaling as a requirement for the proper spatial patterning of branch initiation, as well as a source for active cell migration, has been questioned through the abovementioned work, recent research in our lab and elsewhere has focused on determining the actual physical mechanisms by which Fgfr2b signaling regulates branching morphogenesis. This work highlights the physical regulation of cell and tissue geometries and mechanics, of extracellular matrix (ECM)-driven morphogenesis, and of mesenchymal determinants to branching. There is a growing appreciation for, and understanding of, the interplay between mechanisms under biological control and purely physical and mechanical forces, such as fluid dynamics in the lung. Finally, exciting work in computational and mathematical biology illustrates the search for general models of lung branching morphogenesis.

## Regulation of Cell and Tissue Geometry in the Developing Lung

In the absence of any clear mechanism controlling the observed directed growth of developing lung epithelium, such as filopodial- or lamellipodial-generated invasive migration, investigators have searched for alternative explanations for the epithelial geometries of the lung. This research has focused on cell and tissue biomechanics which lead to epithelial folding (e.g., cell contractility and apical constriction, unequal proliferation between epithelium and mesenchyme, and purely physical properties of mechanical compression and stretching). Once the epithelium buds, it begins to grow in a directed manner and assumes an organized shape. Proliferation of tip cells is higher than those at the cleft or at the stalk, and research on the orientation of the mitotic spindle in branching epithelium sheds light on how directed growth and tube shape is regulated. Finally, the distal tip of the elongating branch begins the process of bifurcation. Here, evidence suggests that the precise location of physical barriers to growth plays a critical role in bifurcation initiation and stability. We will look at these three stages in turn, and highlight the evidence for the role of Fgfr2b signaling in each.

### Bud Initiation

**Figure 2:**
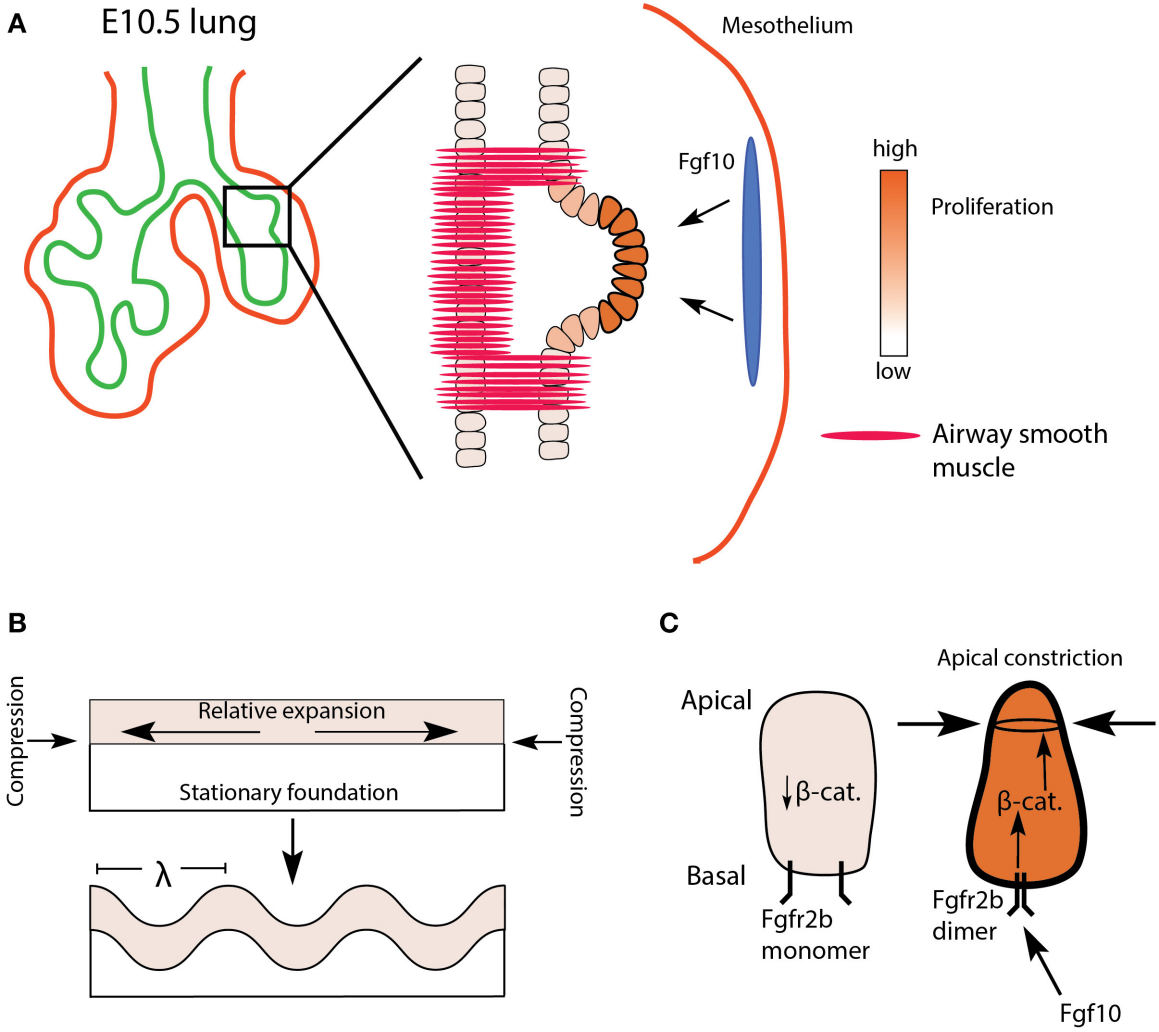
Proposed model of domain branch initiation. **(A)** A domain branch from the left lobe of an E10.5 lung. Differential proliferation is seen shortly after the bud initiates. The blue oval represents localized source of Fgf10 expression. **(B)** Mechanical instability in an expanding sheet (epithelium) constrained by a relatively stationary foundation (mesenchyme) results in a buckling pattern of characteristic wavelength (λ). **(C)** Apical faces of distal epithelial tip cells undergo apical constriction *via* Fgfr2b/β-catenin signaling, supporting the budding process.

It was originally hypothesized that monopodial, or domain branching depended on the localized increase of epithelial proliferation, causing the tissue to bulge outward forming a new bud (Figure 2A). Evidence to support this idea has been conflicting. For example, early *in vitro* studies on mesenchyme-free epithelium cultured in Matrigel demonstrated that localized increases in proliferation *were not* required for bud initiation (Nogawa et al., 1998). However, in live imaging studies on mouse lungs, researchers found that increased proliferation was associated with domain branching but not terminal tip bifurcations (reviewed in Varner and Nelson, 2014). As the evidence for the role of proliferation continues to be assessed, additional mechanisms by which a smooth and uniform epithelial layer would bulge and bud a nascent branch have been considered: apical constriction, unequal proliferation between populations of cells, and physical consequences of mechanical compression and stretching (Varner and Nelson, 2014; Nelson, 2016). One or more of these mechanisms may explain monopodial branching. It has been proposed, for example, that epithelial buckling, which is a purely mechanical phenomenon, may produce the initial pattern of domain budding seen in mammalian lungs. Once established, evidence suggests domain buds undergo apical constriction, a process directed, in part, by Fgfr2b signaling.

Epithelial folding or buckling can arise purely through passive mechanical instabilities, for example, as a result of differential proliferation between two layers of connected tissues, or as a consequence of a tissue being constrained by a surrounding material (reviewed in Varner and Nelson, 2014; Nelson, 2016). *In vitro* cultures of mesenchyme-free epithelium embedded in Matrigel from a number of organs (salivary gland, kidney, lacrimal gland, and intestine), as well as the lung, suggest that a basic mechanical principle underlies the resulting branching of these epithelia. In one such study, Varner et al. (2015) embedded mesenchyme-free lung epithelium from E12 to 13 embryonic mice in varying concentrations of Matrigel. The authors were able to demonstrate that buds formed of characteristic wavelength along the epithelial layer, and that the wavelength of budding was a function of Matrigel concentration. Furthermore, their data suggested that differential proliferation *within* the epithelial sheet (buds proliferating more than stalks) arose only *after* buds formed. In other words, budding arose purely as a consequence of mechanical instabilities arising from a uniformly proliferating sheet being constrained by a surrounding matrix (Figure 2B).

During the initiation of a domain branch, or shortly thereafter, additional processes occur, including increased proliferation at the tip and apical constriction. Apical constriction is mediated *via* actomyosin cytoskeletal reorganization. Early work on actomyosin-mediated contractility, *in vitro*, demonstrated that repressing or enhancing actomyosin activity could, respectively, inhibit or promote branching in lung epithelium (reviewed in Gjorevski and Nelson, 2010). Later, Kim et al. (2013) demonstrated that apical constriction of embryonic chick lung epithelium was necessary to initiate monopodial branching. Furthermore, they found evidence, using an Fgf receptor tyrosine kinase inhibitor, that apical constriction was prevented in the absence of Fgf signaling. However, direct evidence for apical constriction in mouse lung morphogenesis in general, as well as any role played by Fgfr2b signaling in particular, is limited. One recent article published by Fumoto et al. (2017) demonstrated that Wnt signaling regulates the actomyosin cytoskeleton of lung epithelium during the transition from pseudoglandular to canalicular and saccular stages of development. These authors also tested, *in vitro*, whether the Wnt/β-catenin pathway could induce branching in E11.5 mesenchyme-free lung epithelium. They cultured epithelial rudiments in Matrigel with various combinations of low and high doses of rFGF10 with or without an agonist of β-catenin activity (FGF10-CHIR) and found that increased β-catenin activity was associated with increased bud numbers. They also demonstrated that β-catenin was necessary for the apical cytoskeletal organization in these buds. These data suggest that β-catenin acts downstream of Fgf10/Fgfr2b signaling to coordinate cytoskeletal dynamics, perhaps associated with apical constriction (see Figure 2C). This idea warrants further testing. However, whether the *in vitro* model to study apical constriction employed in this paper recapitulates domain branching, *per se*, is questionable; it is unclear if the newly formed buds of mesenchyme-free epithelial rudiments qualify as domain branches.

In a more recent study looking specifically at domain branching in embryonic mouse lungs, Goodwin et al. (2019) suggest, through *ex vivo* studies, that airway smooth muscle (ASM) is required for proper domain branching. These authors first confirmed that ASM forms around primary bronchi in a stereotyped pattern mirroring the pattern of domain branches. Then, through pharmacological, adenoviral, and genetic ablation experiments, this paper found that altering smooth muscle expression around the bronchi affects the stereotypy of domain branching. The authors propose a physical model whereby ASM wrapped around primary bronchi serves to constrain epithelial growth, forcing the epithelium to bud and grow in predictable gaps in the ASM coverage. However, recently the role of ASM in branching morphogenesis has been cast into serious question (Young et al., 2020), and will be discussed more thoroughly in the following section.

Though sparse, the existing literature suggests that monopodial domain bud initiation in mouse embryonic lungs might depend on multiple factors, including epithelial buckling, smooth muscle differentiation and epithelial constraining, as well as epithelial cell apical constriction. Both ASM differentiation and Wnt/β-catenin activity respond to Fgfr2b signaling, and therefore might be critical downstream targets of Fgfr2b-signaling during bud initiation (see below for further discussion of ASM in relation to Fgfr2b signaling). A potential hypothesis is that spatially-patterned domain branching arises from local epithelial mechanical instabilities which are immediately “amplified” by molecular cues and mechanisms (such as apical constriction regulated by Fgfr2b signaling) and “stabilized” by patterns in the surrounding mesenchyme (such as the differentiation of airway smooth muscle) (see Figures 2A,C).

### Coordinated Branch Elongation

**Figure 3:**
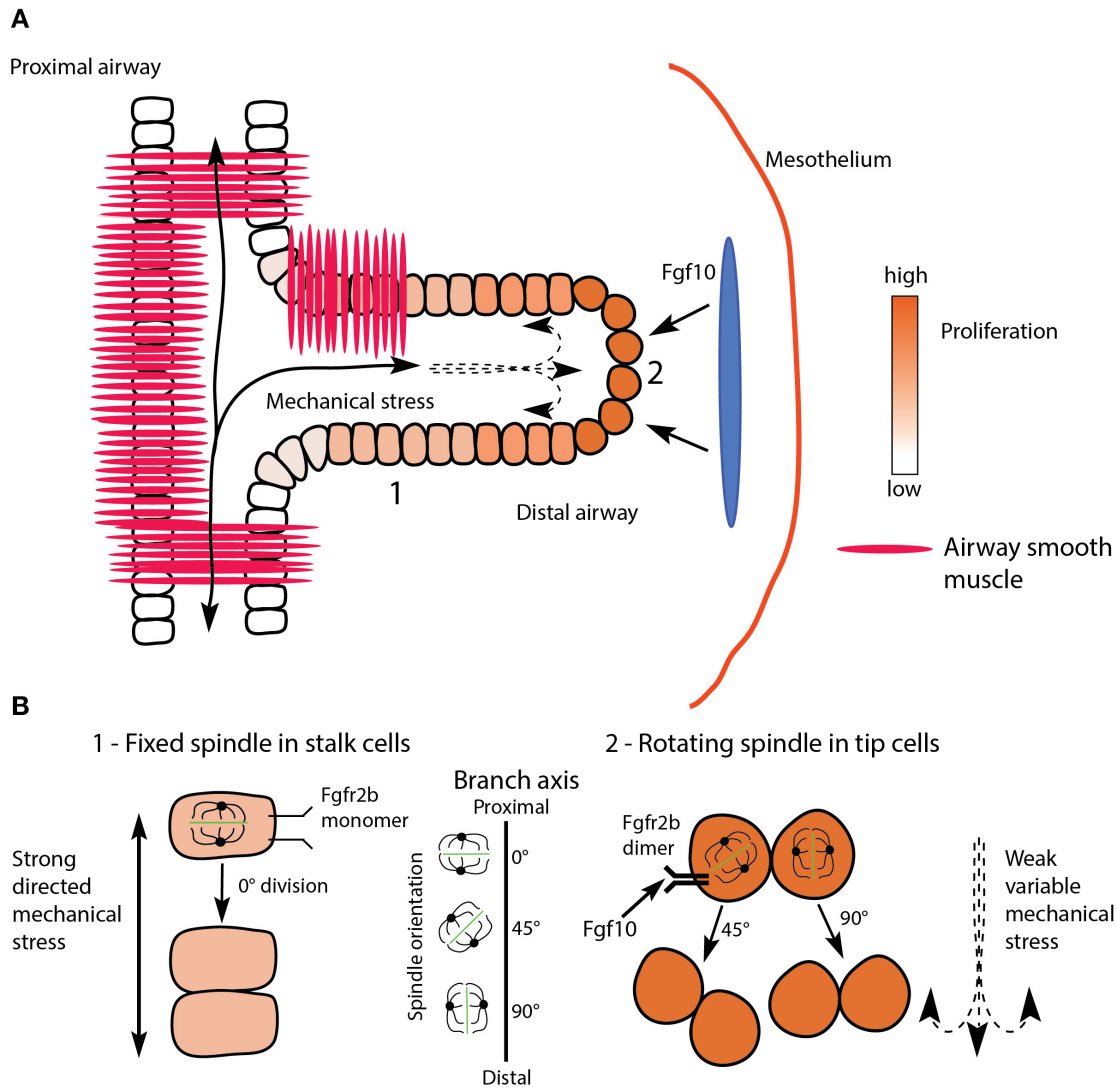
Proposed model uniting mechanical stretching with oriented cell division and Fgfr2b signaling during branch elongation. **(A)** Proximal airways and branch stalks (1) experience strong and directed mechanical stress (solid lines), while distal buds (2) experience weaker, more variable stress (dashed lines). The response of cells to stress is under Fgfr2b regulation. Note how stalk cells show clear apical-basal polarity, whereas tip cells are more rounded. The blue oval represents localized source of Fgf10 expression. **(B)** One of the responses to mechanical stress is spindle orientation during mitosis; proximal stalk cells (1) display a fixed spindle orientation resulting in divisions parallel to the airway long axis, or spindle angles around 0°, while distal cells (2) display rotating spindles resulting in more randomly oriented cell divisions, such as 45° and 90°.

Once a bud forms, differential proliferation is clearly seen. The cells at the tip of the bud proliferate more than those at the cleft or at the stalk (Figure 3A). These tip cells can also be considered as progenitor cells; they are maintained in an undifferentiated and proliferative state characterized, for example, by the differentiation marker Sox9, and by high levels of the proto-oncogene protein N-myc (Okubo et al., 2005; Rockich et al., 2013). More recent work has demonstrated the role of Fgfr2b signaling in the maintenance of these distal epithelial cells in an undifferentiated and proliferative state (Jones et al., 2019a). In this study, Fgfr2b signaling was conditionally inhibited using inducible expression of a soluble form of Fgfr2b in E12.5 and in E14.5 embryonic lungs. It was shown that distal epithelial progenitors lose their Sox9 expression in E12.5 lungs after just 9 h of Fgfr2b inhibition, and while proliferation had yet to be significantly altered in this experiment, a number of genes involved in proliferation, including *Mycn*, were significantly downregulated upon Fgfr2b signaling inhibition. At E14.5, however, proliferation was significantly reduced in distal tip cells after 9 h Fgfr2b inhibition (Jones et al., 2020).

As proliferating cells concentrate at the distal tip of growing airway epithelium, the elongating branches assume a well-defined and organized shape. Therefore, individual epithelial cells must be properly aligned geometrically, relative to the other cells of the tissue. Airway epithelial cells, like many other epithelial cell-types, display apical-basal polarity, with an asymmetry in the distribution of cellular constituents within the cell. On the basal side, epithelial cells attach to the basement membrane through integrin and fibronectin interactions, while on apical lateral sides, cells adhere to their neighbors *via* apical junction complexes, and the apical surface lining the lumen may contain structures such as microvilli. This strict local polarity of cells translates globally to a functioning tissue, and depends on cytoskeletal actin and actin-associated proteins (Winder and Ayscough, 2005), as well as coordinated cell divisions which depend not only on mitotic spindle orientation, but on mechanical forces as well.

Epithelial cells adhere to and communicate with neighboring cells in a tissue *via* tight junctions, adherens junctions, desmosomes, and gap junctions. During morphogenesis, the regulation of cell-cell interactions must be tightly controlled, and early research on this topic has implicated the regulatory role of the planar cell polarity (PCP) signaling pathway (Yates et al., 2010, 2013). The PCP pathway is considered a non-canonical Wnt signaling pathway, and involves a set of core proteins, including planar cell polarity proteins (Vangl) and cadherin EGF LAG seven-pass G-type receptors (Celsr) (reviewed in Vladar and Königshoff, 2020), which were shown to be critical for proper lung branching morphogenesis (Yates et al., 2010). Both *in vivo* and *in vitro* experiments demonstrated that the loss of either *Celsr1* or *Vangl2* resulted in reduced branching, thickened lung mesenchyme, and a disorganized epithelial structure.

Another molecular component involved in PCP, as well as overall cell morphology and migratory behavior, is non-muscle myosin II (NMII) (Vicente-Manzanares et al., 2009). Plosa et al. (2012) found that NMII upregulation constrains cell morphology and orientation, and reduces migratory potential in later stage developing embryos (from E15 onwards). The authors suggest that NMII is likely downregulated in sites of active migration and cell rearrangements.

The majority of research on PCP related proteins and biological control revolves around the non-canonical Wnt pathway, with increasing attention on upstream effectors of that pathway, especially Wnt4 and Wnt5a (Vladar and Königshoff, 2020). Little attention has been paid to regulatory interactions of Fgfr2b signaling on PCP. However, there exists some rudimentary data on the connection between Fgfr2b signaling and epithelial morphogenesis *via* PCP (Jones et al., 2019a, 2020). For example, the PCP gene *Celsr1* was significantly regulated in E12.5 lungs after 9 h Fgfr2b ligand inhibition, while the NMII-associated genes *Myh9* and *Myh10* were regulated after inhibition of Fgfr2b signaling in E14.5 and E12.5 lungs, respectively. More significantly, the putative upstream effectors of PCP, *Wnt4* and *Wnt5a*, are highly regulated by Fgfr2b signaling during pseudoglandular lung development. These early data suggest a direct connection between Fgfr2b signaling and the upstream regulators of PCP, and could provide the impetus for future work on this topic.

Independent of the PCP pathway, Fgfr2b signaling has been shown to directly regulate the stability and turnover of E-cadherin (Cdh1), which is the major protein component of adherens junctions. For example, in early work, Liu et al. (2008) found that mitogen-activated protein kinase (MAPK) p38α signaling, downstream of Fgfr2b, directly regulated the turnover of E-cadherin *in vitro*. Upon inhibition of p38α in cultured lung explants, for example, branching morphogenesis was impaired and Cdh1 expression increased. Furthermore, isolated embryonic lung endoderm ectopically expressing increased Cdh1 failed to branch in culture when compared to normal controls. This finding was later corroborated, *in vivo*, upon Fgfr2b ligand inhibition in E12.5 lungs. In this research, Cdh1 expression in experimental lungs after 9 h Fgfr2b signaling inhibition was drastically increased, and corresponded to disorganized epithelia and lumens which failed to open (Jones et al., 2019a). These results suggest that Cdh1 protein levels are directly controlled by Fgfr2b signaling to allow the cell rearrangements involved in morphogenesis. The molecular events actually taking place at the adherens junction complex in response to Fgfr2b signaling are still to be determined.

In addition to cell polarity and adhesion, individual epithelial cells comprising the branching airway tissue must properly orient during cell division to maintain proper branch shape. To achieve proper orientation, cell divisions show a bias in mitotic spindle angles. Stalk cell divisions are biased to be parallel to the proximal-distal axis of the epithelial layer, while tip orientation is more random, although favoring perpendicular divisions (see following discussion, and Figure 3) (El-Hashash and Warburton, 2011; Tang et al., 2011, 2018; Kadzik et al., 2014).

Literature on the regulatory control of mitotic spindle orientation in relation to branch shape in the developing lung is sparse. Tang et al. (2011) demonstrated that mitotic spindle orientation was controlled by Fgf10-mediated RAS-regulated ERK1/2 signaling in mouse models where ERK1/2 signaling was conditionally activated throughout the epithelium. First, the authors conditionally expressed a mutationally-activated form of RAS (*Kras*), which is an effector of RAS-mediated signaling. They not only found that developing airway tubes lost their normal shape in mutant samples, but that this phenotype was associated with abnormal mitotic spindle angle distributions. Second, the authors conditionally expressed a mutationally-activated member of the RAF family (*Braf*), which acts upstream of ERK signaling. They found similar defects in airway shape as in the first mouse model. Finally, the authors treated *Kras* mutant mice, *in utero*, with a specific inhibitor of the ERK pathway, and found that treated samples had normal tube shapes. Additionally, the authors found that Sprouty proteins, which are negative regulators of Fgf10-mediated ERK signaling, were required to maintain proper mitotic spindle angles, and therefore airway structure. Taken together, this evidence suggests that mitotic angle orientation is more random when ERK1/2 signaling is activated.

In more recent pioneering work from Nan Tang's lab, mechanical force has been shown to play a regulatory role in spindle orientation (Tang et al., 2018). In this research, the authors found that epithelial cells of the developing lung airway adopted either a “fixed-spindle” orientation prior to division, or a random “rotating-spindle” orientation. The spindle orientation a cell adopted was a direct function of cell shape: elongated cells tended to adopt a fixed-spindle orientation, while less elongated cells had rotating spindles (compare elongated stalk cells to rounded tip cells in Figure 3A). The authors also found that cell shape was directly influenced by ERK1/2 signaling, which supports the results of the above-cited study (Tang et al., 2011). Taken together, these two studies strongly suggest that overall lung tube geometry is a function of individual epithelial cell divisions, which are themselves largely determined by cell shape controlled by Fgf10/Fgfr2b-mediated ERK1/2 signaling.

Finally, Tang et al. (2018) also demonstrated, through *ex vivo* manipulation of mechanical stretching on developing lung explants, that increased mechanical force directly regulates the ratio of “fixed-spindle” to “rotating-spindle” cells. As the tension on the epithelial cells increased, so did the ratio of “fixed-spindle” cells to “rotating-spindle” cells, which increased the abundance of cellular divisions parallel to the long axis of the developing airway. In Figure 3B we propose a simplified model uniting the research on epithelial cell shape and spindle orientation downstream of Fgfr2b signaling, with the research on spindle orientation and mechanical stretching (Figure 3B).

Work on spindle orientation due to mechanical stress relates the mechano-sensory responses of cells and tissues to purely physical causes. For instance, it has long been appreciated that intraluminal pressure of branching organs, including the lung, is important for normal morphogenesis. Localized transmural pressure differences can be sensed by cells and tissues and translated into biologically relevant behaviors, such as morphological changes, *via* mechano-sensory mechanisms (Schittny et al., 2000; Bokka et al., 2015b; Nelson et al., 2017). In terms of fluid pressure control of lung branching, Unbekandt et al. (2008) found that cauterizing the tracheas of E12.5 mouse lung explants, thereby increasing the internal intraluminal pressure, resulted in increased rates of branching compared to uncauterized controls. These authors further found that as fluid pressure increased, so too did the expression levels of *Fgf10* and *Shh* mRNA, whereas that of *Spry2* decreased. They concluded that fluid pressure in normal lungs, as determined by epithelial secretions and boundary conditions such as trachea and airway occlusion, regulates branching *via* an Fgf10-Fgfr2b-Spry2 pathway.

Recent papers, employing a combination of modeling and experiment aimed at understanding why occluded lungs show increased branching, hypothesized that localized fluid dynamics create stresses, such as shearing forces, that are potentially sensed by cells and tissues. Intraluminal fluid pressure and flow can be regulated by peristaltic waves, which are produced by ASM contracting in a proximal to distal direction, which partially occludes airways and pushes fluid distally (Schittny et al., 2000; Featherstone et al., 2005; Jesudason et al., 2005). These forces are transduced *via* mechano-sensory mechanisms in epithelial cells undergoing morphogenesis. Furthermore, studies have found that intraluminal flow regulates the transport of morphogens throughout the developing lung (Bokka et al., 2015a,b, 2016; George et al., 2015). For example, Bokka et al. (2015a) modeled and studied airway peristalsis resulting from airway smooth muscle contractions, and concluded that flow rates and shearing forces, as determined by peristaltic activity, may help inform epithelial cells of their geographic location within the airway network. This geographic information may induce these cells to respond accordingly, by, for example, orienting their cell divisions, or forming thickened epithelial sheets in the case of proximal bronchi. In a related study from the same group (George et al., 2015), computational modeling of tissue stretch and solute transport in a 3D model of an E12.5 lung predicted that morphogen concentration in the mesenchyme will not only increase as a result of increased intraluminal fluid pressure, but also that morphogen flux (defined as the distribution patterns of morphogen binding and transport) will increase around distal tips. These predictions correspond nicely with the findings of Unbekandt et al. (2008), and demonstrate the potential predictive power of computational modeling of complex biological phenomena.

**Figure 4:**
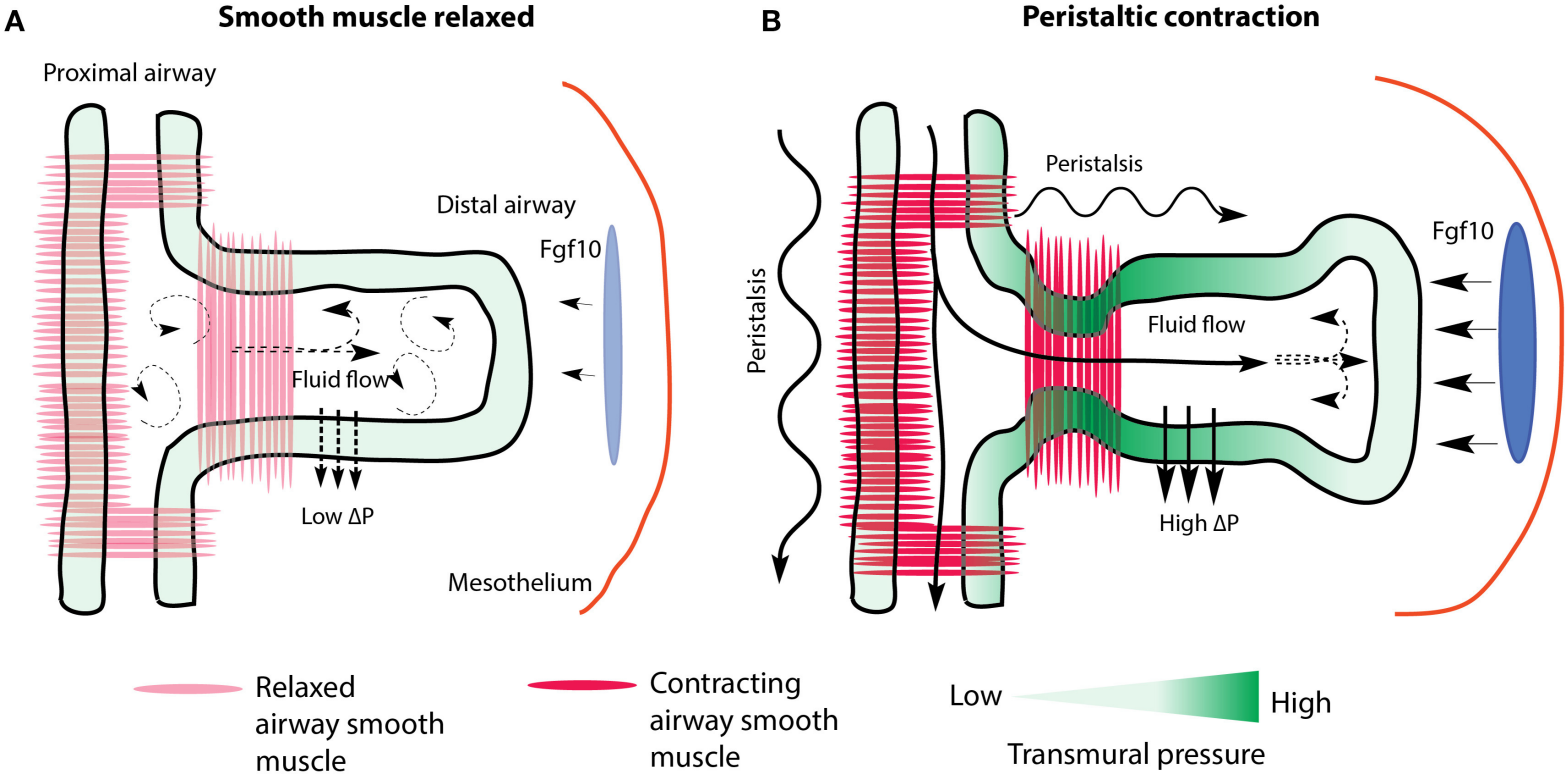
Prevailing model of the regulation of fluid dynamics by airway smooth muscle during branch elongation. **(A)** When smooth muscle is relaxed luminal fluid flow is weak and transmural pressure (ΔP) is low and non-localized. Mesenchymal Fgf10 flux is reduced. **(B)** During peristalsis, luminal fluid flow increases, creating high localized transmural pressure, which directs branch elongation, as well as increases mesenchymal Fgf10 concentration and flux around the tip. Dark and light blue ovals represent localized sources of high and low Fgf10 expression, respectively.

Finally, a study by Nelson et al. (2017), which creatively employed micro-fluidic chambers to experimentally manipulate luminal and pleural pressures of E12 lungs, *ex vivo*, not only confirmed that transmural pressure regulated the rate of branching morphogenesis, but also that branching was synchronized with airway smooth muscle contractions. In their study, as transmural pressure increased, the time interval between peristaltic contractions of proximal smooth muscle decreased, which resulted in increased rates of branching (these studies are summarized graphically in Figure 4). It is critical to mention here, however, that even more recent data on ASM peristalsis casts serious doubt on the prevailing interpretation, which is, to a large extent, based on *in vitro* research. *In vivo* studies conducted by Young et al. (2020), reported the consequences of inactivating *Myocardin* (*Myocd*) in early stage embryos. *Myocd* encodes a transcription factor necessary for ASM differentiation. Embryonic lungs were then assessed at different time points throughout the pseudoglandular stage of lung development. It was found that lungs branched normally, albeit with branches of smaller diameter, even in the absence of ASM peristalsis.

Clearly more work is needed to reconcile the competing interpretations of the role of ASM in lung branching. Nevertheless, these studies reveal a new avenue of research, one which unites physical regulation of morphogenesis, such as fluid dynamics related to peristaltic contractions of airway smooth muscle cells, to molecular regulation, such as cell shape and behavior modulated by Fgfr2b-ERK1/2 signaling. It will be interesting to investigate, for example, if the more dynamic orientation of tip cells, relative to stalk cells, in the developing epithelium is a direct consequence of lower mechanical stress and higher Fgfr2b signaling at the tip. It will also be interesting to identify proteins involved in mitosis that are directly regulated by Fgfr2b signaling. One candidate is Nubp1, which is highly expressed in the distal epithelial tip progenitors of developing lungs, and which is required for proper branching (Schnatwinkel and Niswander, 2012). This protein is involved in centrosome localization and microtubule dynamics in the cell. It also appears to be transcriptionally regulated by Fgfr2b signaling during pseudoglandular development (Jones et al., 2019a, 2020).

### Branch Tip Bifurcation

After a period of growth, elongating branches arrest, and the distal tips undergo planar or orthogonal bifurcation (Metzger et al., 2008). In general, tip bifurcation involves four steps: (1) branch arrest and tip dilation, where the branch stops its directional growth and the distal lumen swells to form a bulb; (2) tip flattening, where the bulbous tip assumes a flattened distal edge; (3) cleft initiation, where a cleft forms at the midline of the flattened tip; and (4), cleft deepening and sister branch elongation, where the newly formed branches restart the iteration of elongation and eventual bifurcation (Kim et al., 2015).

There are numerous molecular events which occur in the distal epithelium and surrounding mesenchyme during branch arrest and bud dilation. These include, in addition to the well-established crosstalk between Fgf10 and Shh, an increase in *Spry* gene expression in the epithelium, which antagonizes Fgf10 signaling, thus contributing to bud arrest. The localized expression domain of Fgf10, in turn, splits into two domains, which lie adjacent to the newly forming branches (reviewed in Warburton et al., 2005).

There are purely physical mechanisms by which a single branch can bifurcate, one of which is the establishment of a barrier to the directional growth of the branch. Once encountered by the growing branch, the barrier causes the branch tip to cleft and bifurcate, much like a river splits around a boulder. Researchers have begun to identify these barriers in the developing lung mesenchyme and extracellular matrix (ECM). For example, De Langhe et al. (2005) found that inhibition of canonical Wnt signaling in the distal region of the developing lung by epithelial secretion of dickkopf (Dkk1) directly controlled fibronectin (FN) deposition in the ECM. These authors showed, through inhibition and rescue experiments, that FN is a key component of cleft formation in the lung; in the absence of FN, clefts failed to form in branching airway epithelium, distal buds assumed a dilated phenotype, and the total number of branches was reduced. This finding mirrors earlier evidence that showed FN associates with cleft formation in developing salivary glands (Sakai et al., 2003), and is supported by later work in the salivary gland and lung which demonstrated that FN activates the regulatory protein Btbd7 in the epithelial cells of clefts (Onodera et al., 2010). In this work, Btbd7 was shown to regulate epithelial cell scattering and motility, thus providing a mechanistic link between FN deposition and the epithelial cell rearrangements involved in bifurcation.

A second barrier to directional growth was posited through work on airway smooth muscle cells (ASMC), wherein small localized pools of ASMC were discovered to form in distal mesenchyme adjacent to epithelial buds (Kim et al., 2015). In this study, alpha-smooth muscle actin (α-SMA) RFP reporter mice were used to label ASMCs, and time-lapse imaging of distal buds in E12 lung explants was employed to follow the development of a bud through the four stages of bifurcation (growth arrest, bud flattening, cleft formation, and bifurcation). A fascinating finding from this work was that ASMCs were found to localize in the mesenchyme adjacent to future cleft sites *before* the cleft formed and bifurcation commenced. Furthermore, the authors were able to demonstrate that proper ASMC differentiation and mesenchymal patterning is essential for proper epithelial branching; clefts failed to form in the absence of ASMCs, and terminal buds eventually adopted a “buckled” phenotype.

As mentioned in the previous section, the recent paper by Young et al. (2020) is problematic for the aforementioned published studies and interpretations of ASM regulation of branching. In this paper, wherein ASM was prevented from differentiating *in vivo*, the authors found that branches apparently bifurcated normally in the absence of smooth muscle at cleft sites. Whether clefting is still possible because of other barriers, such as fibronectin, or whether it depends on entirely different mechanisms, is still left to be determined.

How ASMCs properly pattern in the distal mesenchyme and their precise interactions with the ECM and distal epithelium remain topics of research. It is known that ASMC are derived, in part, from Fgf10-positive cells in the submesothelial mesenchyme (SMM) during lung development (Mailleux et al., 2005; El Agha et al., 2014, 2017). These ASMC progenitors migrate proximally and differentiate to *bone fide* ASMC. This process is controlled in part by Fgf9 and Shh. Fgf9 is secreted by the mesothelium until E13.5 and by the distal epithelium afterwards (del Moral et al., 2006). Fgf9 signaling increases Fgf10 expression in the mesenchyme, as well as inhibits ASMC differentiation distally and ASMC expansion proximally (El Agha et al., 2017). In addition, epithelial Shh expression, itself controlled by Fgf10 in the mesenchyme, has been shown to induce ASMC differentiation in the surrounding mesenchyme (Weaver et al., 2003; White et al., 2006). Whether if, and how, the crosstalk among Fgf9, Fgf10, and Shh patterns ASMC distribution adjacent to nascent bud clefts remains to be investigated.

**Figure 5:**
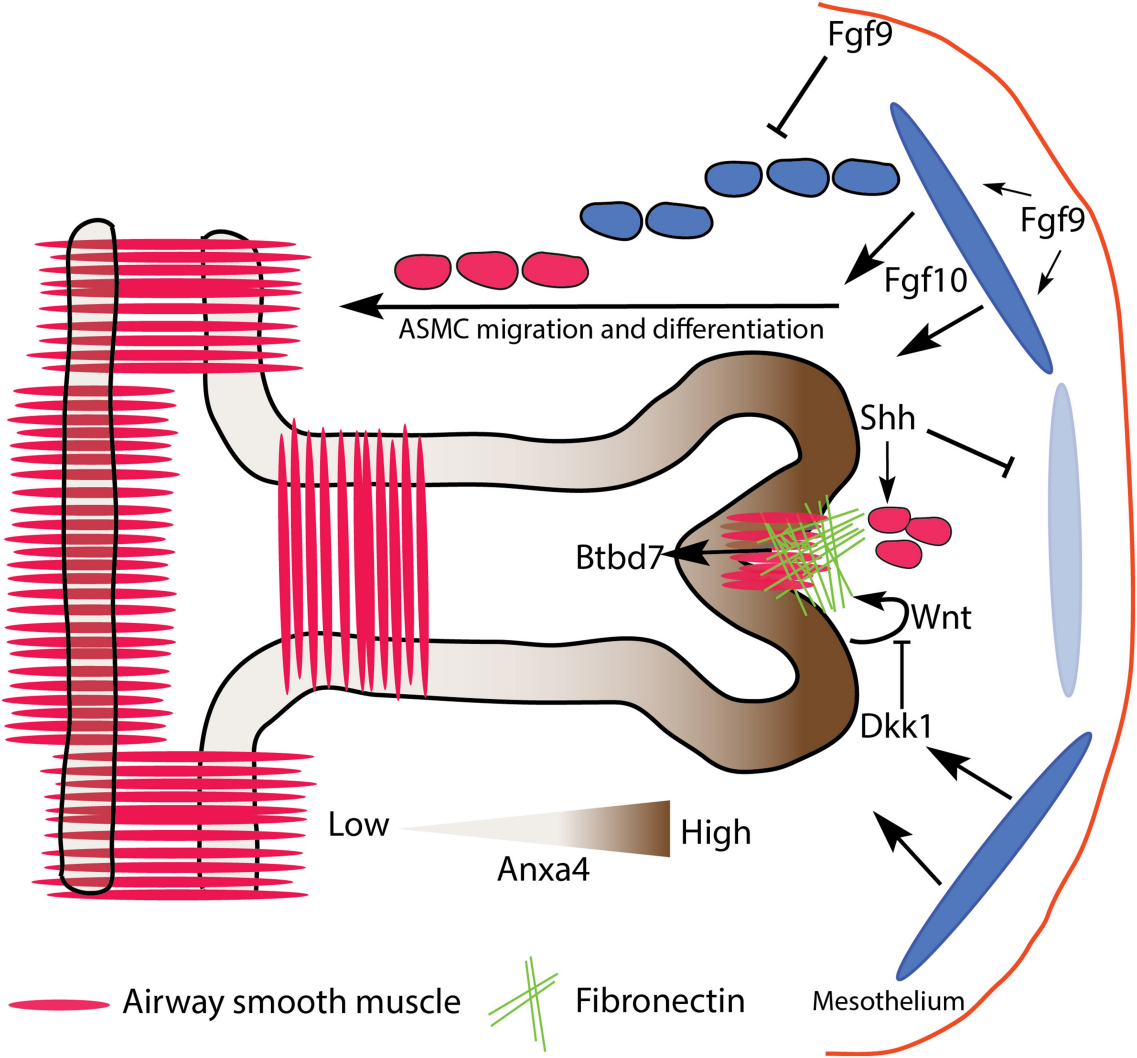
Proposed model of mesenchymal barriers to epithelial growth resulting in bifurcation. A combination of localized fibronectin and airway smooth muscle (ASM) expression forms a physical barrier to expanding branches, resulting in tip cleft formation and bifurcation. ASM partly derives from Fgf10 expressing progenitors, which are regulated upstream by Fgf9, and downstream by Shh. Fibronectin deposition is regulated by Dkk1/Wnt signaling downstream of Fgfr2b, and upregulates the expression of Btbd7 in the cleft. Anxa4 marks tip progenitor cells, and is involved in cell migratory behavior. Dark and light blue ovals represent localized sources of high and low Fgf10 expression, respectively.

Regardless of the controversy surrounding ASM, once a cleft forms, bifurcation commences. During this stage, ASMC expansion is seen, not only in the cleft, but also in the neck of the growing buds. This smooth muscle wraps around the cleft and the neck as the daughter branches elongate, likely providing support and directional cues to the growing epithelium. Also during this stage, progenitor cells marked by Annexin A4 (Anxa4) expression actively migrate to the daughter bud tips, and help maintain those cells in an undifferentiated progenitor status (Jiang et al., 2018). This process is regulated by Fgfr2b ERK1/2 signaling (these ideas are summarized in Figure 5).

## Extracellular Matrix Remodeling

Until now we have looked primarily at the direct regulation of epithelial tissue through bud initiation, branch elongation, and tip bifurcation. Here, it is critical to be reminded of a basic fact: that the mechanisms discussed thus far largely overlap and interact throughout a branching event; fluid dynamics, for instance, are at play at each stage. It is also critical to discuss the more indirect and passive regulation of airway branching, which depends on extracellular matrix (ECM) remodeling.

The signaling ligands and their receptors responsible for the molecular control of branching morphogenesis interact in a dense milieu of extracellular components comprising the ECM. The ECM is a network of molecules secreted by and connecting the mesenchymal and epithelial compartments of an organ. It is composed of numerous families of proteins, including laminins, fibronectin, collagen, matrix metallopeptidases (MMPs), and glycoproteins (ref. from Patel et al., 2017). The ECM provides an adhesive foundation for the epithelial tissue layer, contains molecules that either act as support or barriers to airway branching, and also facilitates and modulates the diffusion patterns and binding affinities of signaling molecules. For these reasons, understanding the regulation of the ECM during branching morphogenesis is of critical importance.

### Heparan Sulfate

Heparan sulfates (HSs) are linear glycosaminoglycans composed of repeating sulfated disaccharides which are attached to protein backbones in the Golgi apparatus. These HS-protein complexes are known as HS proteoglycans (HSPGs), and are found on cell surfaces as well as in the ECM during organogenesis and homeostasis. After HSPG synthesis, HS chains can be modified or removed by enzymes, which drastically expands the affinity of HS for different molecules, and increases the biological roles played by HS. HS is indispensable for normal lung physiology and function, and has been implicated in the regulation of numerous biological processes, including branching morphogenesis (reviewed in Haeger et al., 2016; Patel et al., 2017).

In *in vitro* experiments, where either HS biosynthesis or post-synthesis modification has been impaired, detrimental effects on lung branching have been observed (reviewed in Patel et al., 2017). For example, digestion of heparan sulfate in mesenchyme-free E11.5 lung explants resulted in the death of the lung similar to that seen in the absence of Fgf10, while chemical disruption of endogenous HS sulfation gradients drastically reduced Fgf10 binding (Izvolsky et al., 2003a,b). From this research, HS was shown to be a powerful and necessary cofactor for Fgf signaling during lung organogenesis. Furthermore, it was later found that Fgf10, but not Fgf7, binds with high affinity to HS (in particular, HS with 6-O-sulfated residues) (reviewed in Ornitz and Itoh, 2015; Zinkle and Mohammadi, 2019).

Given the seemingly necessary interaction of Fgf10 and HS for branching *in vitro*, subsequent *in vivo* studies using mouse models to conditionally inactivate genes involved in HS biosynthesis and sulfation have led to surprising results. For example, in one study where *heparan sulfate 6-O-sulfotransferase-1* (*Hs6st1*) was genetically inactivated, defects on the developing lung were not reported (Habuchi et al., 2007), even though late embryonic mortality was observed. A later study employing a similar model found that inactivation of *Hs6st1* had no effect on organogenesis, and that impacts on lungs were only seen in postnatal and adult mice in the form of enlarged airspaces and alveolar defects (Izvolsky et al., 2008). Hs6st1 is one of a family of three proteins (Hs6st1-3) which sulfate the 6-O residue of HS, and is most highly expressed in the developing lung at distal epithelial tips. Loss of Hs6st1 was predicted to impact branching morphogenesis due to the affinity of Fgf10 for 6-O-sulfated HS. The lack of any clear phenotype in the embryonic lungs analyzed in these studies can perhaps be explained by compensatory effects of the other two Hs6st enzymes, by compensation through the lower affinity binding of Fgf10 to 2-O-sulfated HS, or by an additional HS-independent mechanism.

A more recent publication has further questioned the role of Fgf10-HS interactions during lung branching morphogenesis by suggesting that HS regulation of Shh signaling is of key importance (He et al., 2017). In this study, *exotosin glycosyltransferase 1* (*Ext1*), which encodes a protein necessary for HS polymerization, was conditionally ablated in the epithelium of developing lungs. The authors found that mutant lungs had significantly reduced branch numbers, along with dilated distal tips. They found that levels of Shh protein were not altered in mutants, but that Shh signaling was disrupted, an effect which could be rescued, *in vitro*, by using smoothened agonists. Interestingly, Fgfr2b signaling, which was predicted to be decreased in the absence of epithelial HS, was actually found to be increased. The domain of Fgf10 expression in the mesenchyme expanded in mutant lungs, along with phosphorylated ERK expression in the epithelium. The authors argue that this overexpression of Fgf10 explains the dilated branch tips, and is a direct consequence of disrupted Shh signaling. Finally, the paper concludes by demonstrating that HS serves as an important repository for Shh protein, and is required for the production of the biologically active form of Shh involved in Shh-Fgf10 crosstalk.

These *in vivo* studies, though few in number, demonstrate that the role of Fgf10-HS interactions during lung branching morphogenesis may not be as vital as *in vitro* studies suggest, although clearly more detailed studies are required. What does emerge from this work, however, is the regulation of branching *via* Shh-HS dependent signaling. Furthermore, it is likely that the spatio-temporal distribution of HS in the developing lung is as important as the distribution of the signaling ligands for which it serves as a cofactor, and as such, should be the focus of further *in vivo* studies on branching morphogenesis (Thompson et al., 2010).

In addition to HS, the role of secreted laminin-related netrins during branching morphogenesis has received limited, yet increasing, attention in the literature (Murray, 2017). Netrins 1 and 4, for example, have been shown to be secreted by developing epithelium, especially around the stalk and neck of branches, and have been implicated in the inhibition of Fgfr2b-ERK1/2 signaling-regulated tissue morphology (Dalvin et al., 2003; Liu et al., 2004). As is the case with HS, the regulation of and by netrins during branching morphogenesis needs further elucidation.

### Basement Membrane Dynamics and ECM Fluidity

The basement membrane (BM) is a specialized ECM structure which separates the airway epithelium from, and attaches it to the surrounding mesenchyme and interstitial ECM. It is composed of proteins from a number of families, including laminins, integrins, collagens, fibronectin, and heparin sulfates. This diversity of proteins drastically increases the spatial and temporal properties of the BM during branching morphogenesis, conferring an additional level of regulatory potential. Laminins, for example, are heterotrimeric glycoproteins composed of an alpha, beta, and gamma subunit, and comprise a large family of 15 isoforms, with different isoforms conferring different properties to the BM (Nguyen and Senior, 2006). The laminin α5 (Lama5) subunit, for instance, is involved in lobar septation, while Lama1 has been implicated in proper branching morphogenesis (Nguyen et al., 2002; Nguyen and Senior, 2006). It has been suggested that Fgfr2b signaling regulates the expression of *Lama1, Lama3*, and *Lamc2* gene expression, as well as Lama1 protein expression (Jones et al., 2019a). In the latter, immunofluorescence data revealed that Fgfr2b inhibition resulted in increased Lama1 expression in the BM of airway epithelium and of mesothelium in E12.5 lungs. These results are in line with earlier work on branching morphogenesis, which found that BM thinning is required for branch elongation, and which coincides with increased proliferation of the growing epithelium (reviewed in Moore et al., 2005; Gill et al., 2006).

Research on basement membrane remodeling has shown that thinning is a function of mechanical stresses in the epithelium, which exert tractional forces on the BM and which are resisted by the surrounding ECM (Ingber, 2003; Moore et al., 2005). According to one hypothesis (Moore et al., 2005), during branching morphogenesis, domains of mechanical instabilities arise in the BM as a result of ECM degradation, and the BM thins. Research suggests that the tension shifts experienced in these domains are sensed by the adjacent epithelial cells, resulting in cytoskeletal, and thus cellular, reorganization. This reorganization is partly controlled by the small-GTPase Rho signaling through the Rho-associated kinase pathway (ROCK). Furthermore, the thinner BM and reorganized epithelium increase access to and binding with mitogens and other growth factors, thus resulting in the observed increased proliferation of these epithelial cells (Moore et al., 2005).

Balanced extracellular matrix degradation is achieved through offsetting activity between various species of matrix metalloproteinases (MMPs) and specific tissue inhibitor of metalloproteinases (TIMPs) (Bonnans et al., 2014; Arpino et al., 2015). Near the tips of growing buds, relative MMP activity is higher than in the cleft or at the stalks, where relative TIMP activity is higher. In studies where MMP is either over-expressed or repressed, branching morphogenesis is inhibited (Gill et al., 2006). It is likely that the coordinated effects of MMPs and TIMPs provide the spatial information for patterned basement membrane thinning, and thus directed branch elongation.

ECM protease activity also relates to the idea of tissue fluidity, which conceptualizes the epithelium and surrounding mesenchyme as fluid structures, each with measureable and characteristic fluid dynamic properties, such as surface tension, viscosity, and compressibility (Manning et al., 2010; Bi et al., 2016). Localized increase in fluidity (in other words, a decrease in viscosity) can occur as a result of changes in relative ECM protein constitution (more or less collagen relative to other proteins, for example), an increase in MMP activity, or a change in the shape and/or number of mesenchymal cells. As already discussed, MMP activity is higher around distal tips relative to the cleft or the stalk. Recent studies have shown, in embryonic chicken lungs, that MMP activity adjacent to developing airway branches decreases ECM fluidity, and is required for branch extension (Spurlin et al., 2019). This is a result which supports evidence from other species and organs, including kidney and lung branching in the mouse (Rutledge et al., 2019).

One of the ECM proteins related to decreased tissue viscosity and remodeling (especially of the BM), is tenascin-C (TNC) (Spurlin et al., 2019). Tenascin-C is a large and evolutionarily conserved protein, with many variations based on splicing and post-translational modifications, and is ubiquitously expressed in multiple organs during development, including the lung (Midwood et al., 2016). TNC interacts with the BM, and is associated with BM thinning, likely through interactions with fibronectin (see references in Giblin and Midwood, 2015). TNC also serves as a binding partner to many ligands and morphogens diffusing through the ECM (De Laporte et al., 2013). It is unsurprising, therefore, that TNC has consistently been shown to be critical for proper lung branching morphogenesis (Roth-Kleiner et al., 2004; Gebb et al., 2005; Spurlin et al., 2019; Gremlich et al., 2020).

Given the importance of TNC to proper organogenesis, the literature is sparse on the regulatory pathways controlling TNC expression during development. In the context of Fgf signaling, it has been shown, for example, that TNC is regulated by Fgf4 in chick limb tendons, as well as by Fgf1 in the CNS (Edom-Vovard et al., 2002; Suzuki et al., 2002); whereas a more recent study has shown that TNC binds to many Fgf family member ligands (De Laporte et al., 2013). However, very little is known about the role and regulation of TNC during mouse lung development.

**Figure 6:**
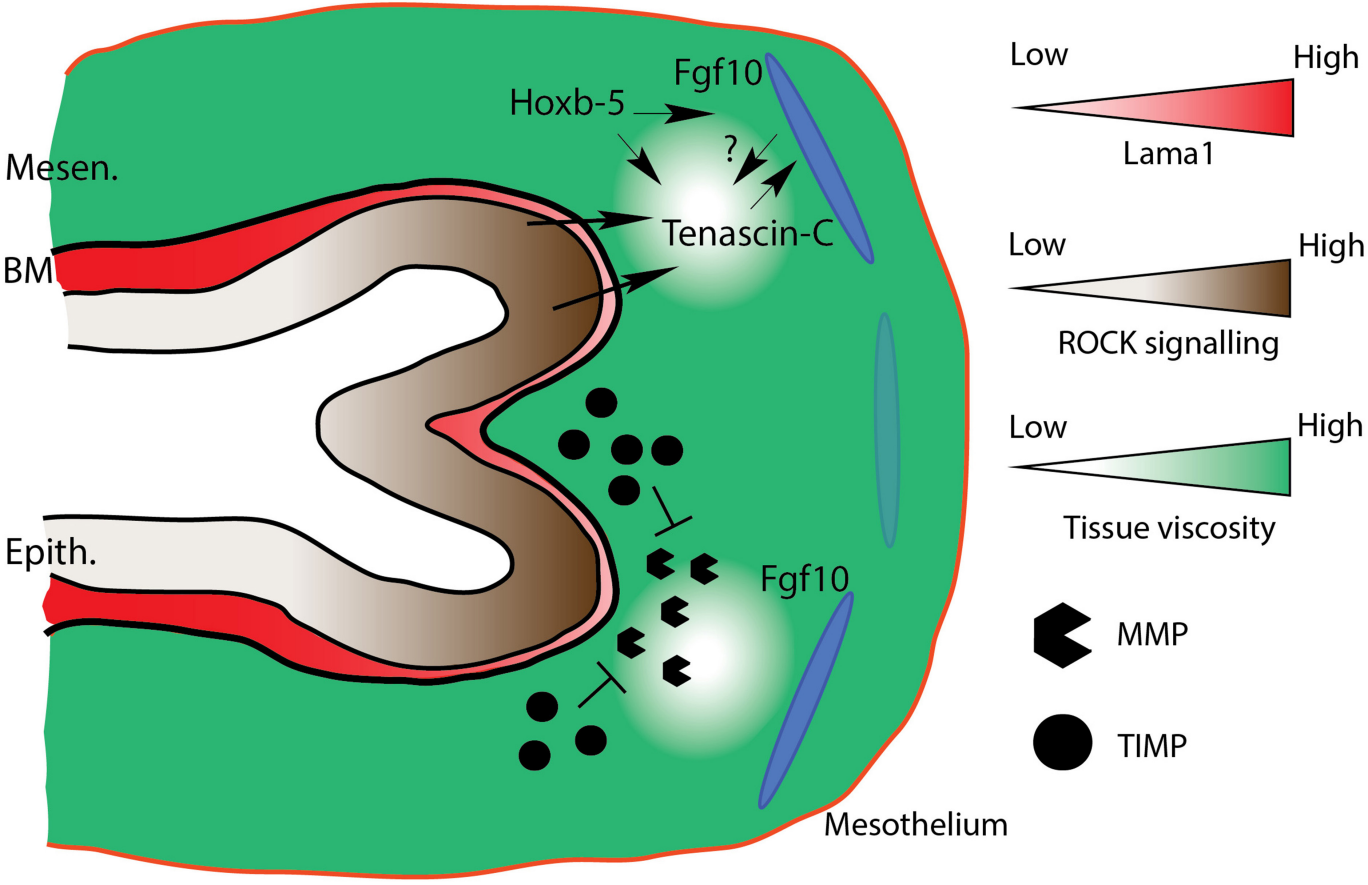
Model of ECM remodeling and tissue fluidity. Mesenchymal tissue fluidity increases around distal bud tips in response to increased MMP activity. Increased fluidity increases expression of tenascin-C (TNC) around distal tips. TNC regulates mesenchymal Fgf10 activity, and is perhaps itself regulated by Fgf10. Hoxb-5 regulates TNC expression, and also the domain of Fgf10 expression, either directly, or indirectly *via* TNC regulation. Increased ECM fluidity corresponds to basement membrane thinning (e.g., lower Lama1 expression), which results in increased ROCK signaling-directed cellular rearrangements. Mesen., Mesenchyme; BM, Basement membrane; Epith., Epithelium. Dark and light blue ovals represent localized sources of high and low Fgf10 expression, respectively.

One study has posited that homeobox (*Hox*) genes, coding for “master regulatory” transcription factors during embryogenesis, may interact with the expression of Fgf10 and tenascin-C during branching morphogenesis of the lung (Volpe et al., 2007). In this study, where *Hoxb-5* was knocked down in cultured fibroblasts and in whole lung explants using small-interfering RNA technology, it was found that loss of *Hoxb-5* expression resulted in a decrease in TNC and *Fgf10* gene expression. It was also found that TNC protein was drastically reduced in *Hoxb-5* knockdowns, whereas a trend to reduced *Fgf10* was reported, corresponding to reduced branching and impaired airway morphometry. Finally, it was found that the spatial distribution of *Fgf10* expression in *Hoxb-5* knockdowns was expanded, with a more diffuse mesenchymal expression observed. The authors posit that the spatial restriction of Fgf10 is either directly regulated by *Hoxb-5* expression, or indirectly by *Hoxb-5* regulation of TNC. This early research highlights the need for ongoing work on ECM remodeling and tissue fluidity in mammalian lung morphogenesis to identify the molecular regulatory pathways at play during these processes (see Figure 6 for a model of ECM remodeling).

## The Other Lung Trees: Blood Vessels and Nerves

**Figure 7:**
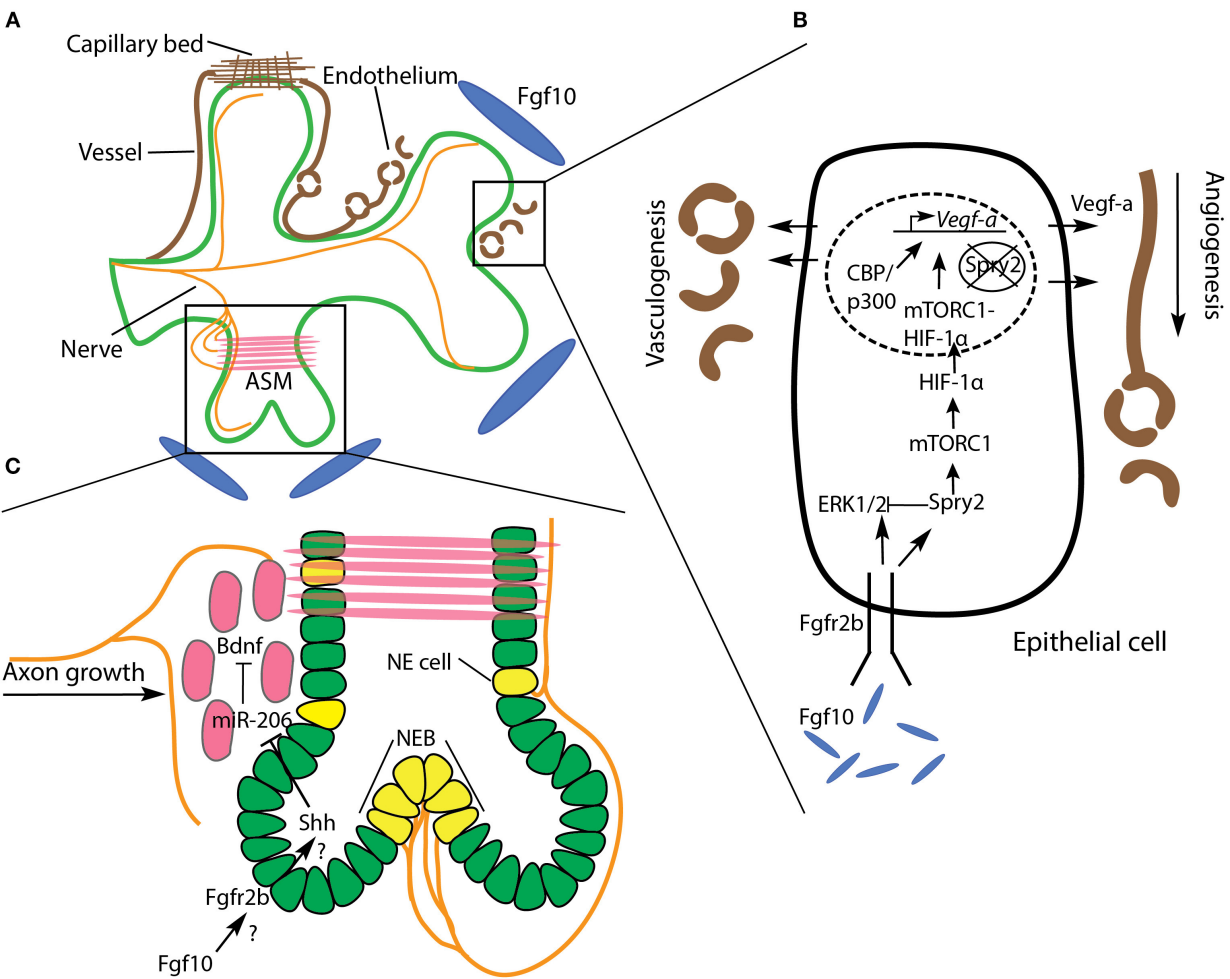
Relationship of the vasculature, nerves, and airway systems during branching morphogenesis. **(A)** The development of branching airway epithelium (green) is tightly coordinated with vascularization (brown) and depends on nerves (orange) innervating surrounding airway smooth muscle (ASM). **(B)** A simplified airway epithelial cell is illustrated to show the role of Fgf10 signaling on vasculogenesis and angiogenesis in the adjacent mesenchyme. Fgf10/Spry2 signaling promotes *Vegf-a* expression *via* mTORC1-HIF-1α. See text for details. **(C)** Nerves not only innervate ASM, but neuroendocrine (NE) cells and neuroepithelial bodies (NEB) as well. These rare cells have been implicated in the branching process, though little is known about the underlying mechanisms. ASM progenitors (red) guide axonal growth toward regions of ASM formation by releasing Bdnf. Bdnf is downregulated by miR-206, which is itself downregulated by Shh secreted from the epithelium. We speculate that this regulatory axis lies downstream of Fgf10 signaling.

The airway tree, of course, is not the only branching structure found in a functional mammalian lung, which also includes a complex network of blood and lymphatic vessels, as well as nerves. A comprehensive review of the development of these other pulmonary trees is beyond the scope of the current paper. However, a complete picture of epithelial branching must account for the role played by these other pulmonary structures in airway morphogenesis. In this section, we briefly discuss the blood vasculature and the nerve network in relation to airway branching, and consider the role of Fgfr2b signaling therein (see Figure 7).

### The Vascular Network

Vasculogenesis is the process by which initial blood vessels are formed, *de novo*, from mesoderm-derived endothelial precursors (reviewed in Ochoa-Espinosa and Affolter, 2012). These initial vessels form the scaffolding for subsequent sprouting angiogenesis, where, like that of the airway tree, a complex, largely stereotyped, vascular network is produced. A functional lung must optimize gas exchange between the airways and the blood vasculature system. It is unsurprising, therefore, that the arborized architecture of pulmonary blood vessels and arteries mirrors the airway tree along the proximal to distal axis, where, at distal tips, a capillary plexus enwraps each alveolus (see Glenny, 2011; Zepp and Morrisey, 2019) (Figure 7A).

A key signaling ligand involved in vasculo- and angiogenesis is vascular endothelial growth factor A (Vegf-a). This ligand is secreted by airway epithelium shortly after lung bud initiation, and signals to its cognate receptor in the mesenchyme, to commence vasculogenesis and angiogenesis concomitantly with airway morphogenesis. When even a single *Vegf-a* allele is knocked-out in mice, embryos exhibit a lethal phenotype at the earliest stages of lung development (E9.5-E10.5). Furthermore, isoforms of the hypoxia inducible factor (HIF) family of transcription factors are known regulators of *Vegf-a* gene expression. For example, *Hif-2*α-deficient mice die from respiratory failure at birth, and the expression of Vegf-a in *Hif-2*α-null mice is drastically reduced (reviewed in Warburton et al., 2005).

Control of Vegf-a expression in the epithelium is tightly coordinated during airway morphogenesis to ensure the concomitant development of the adjacent vascular network. Work by Stephen Land and colleagues has uncovered some of the regulatory mechanisms by which Vegf-a expression is controlled in the airway epithelium (Scott et al., 2010; Land et al., 2014; Walker and Land, 2018). Working predominantly on rat models, with supporting evidence from mouse systems, these authors initially demonstrated that Fgf10/Fgfr2b/Spry2 signaling activates a rapamycin complex 1 (mTORC1)-HIF-1α complex which drives Vegf-a production and secretion from the airway epithelium (Scott et al., 2010; Land et al., 2014). This finding illustrates the coordinated development of pulmonary airways with adjacent vasculature downstream of Fgfr2b signaling, a relationship which has been independently corroborated in our lab's work on Fgf10 heterozygous mouse models, wherein varying levels of *Fgf10* expression have a pronounced effect on embryonic pulmonary vasculature (Chao et al., 2017, 2020).

In a more recent study, and prompted by the discovery that Spry2 is present in the nuclei of branching airway epithelium, Walker and Land (2018) investigated whether nuclear Spry2 is also involved in the Fgf10/Spry2/mTORC1 regulation of *Vegf-a* expression. Here they demonstrated that nuclear Spry2, in the absence of Fgf10/Fgfr2b signaling, binds to the promoter region of *Vegf-a*, preventing gene transcription. However, sustained Fgf10/Fgfr2b signaling, which activates the mTORC1-HIF-1α complex, was found to clear nuclear Spry2 expression. Once cleared from the nucleus, the mTORC1-HIF-1α complex formed a stable association with CBP/p300 at the *Vegf-a* promoter region, driving *Vegf-a* expression. Thus, Fgf10 signaling regulates *Vegf-a* expression, *via* mTORC1-HIF-1α, by modulating cytoplasmic and nuclear Spry2 (Figure 7B).

While the majority of research linking airway and vascular morphogenesis highlights the role of ligands secreted from the epithelium in inducing and patterning vasculo- and angiogenesis, it should be noted that an instructive role of the vascular tree on airway morphology also exists. For example, in both *in vivo* and *in vitro* experiments where pulmonary vasculature was ablated during branching morphogenesis, it was discovered that airway branch stereotypy was drastically impaired (Lazarus et al., 2011). In this study, after vascular ablation, although ectopic branching was seen, airways seemed to branch at nearly normal rates. However, the geographic patterning of branches was affected, especially of those branches bifurcating out of the plane of the parent branch. It was suggested that the spatial patterning of Fgf10 in the mesenchyme was affected by the loss of vasculature, and thus the downstream regulators of branching (Shh and Spry2), were imprecisely expressed. Taken together, research to date suggests that Fgf10-regulated Vegf-a expression in the airway epithelium induces vasculo- and angiogenesis in the adjacent mesenchyme, which in turn regulates the stereotypy of the branching airways, all of which promotes the tight coordination of these two branching networks required for optimal gas exchange.

### Nerve Network

Shortly before pseudoglandular lung development proper begins in the mouse (around E11), in addition to the vagus nerve and its processes, the lung contains neuronal precursors derived from neural crest cells. A day of embryonic development later (E12), these precursors form nerve bundles in proximal regions which run along the bronchi following a path of differentiating airway smooth muscle. As pseudoglandular development continues, the nerves extend distally as far as branching tips, while also projecting some fibers into the surrounding mesenchyme (Tollet et al., 2001; Burns et al., 2008; Bower et al., 2014). This network of pulmonary nerve tissue is part of the parasympathetic nervous system, and is primarily involved in airway and vascular smooth muscle innervation.

In studies where parasympathetic innervation has been perturbed, airway branching was negatively affected. For example, Bower et al. (2014), employing a unique laser technique to specifically ablate pulmonary nerves, demonstrated that nerve tissue ablation led to reduced branching, not only in vertebrates (mouse), but in invertebrates as well (*Drosophila*), and that this effect was independent of neurotransmission. Furthermore, in mouse models of congenital diaphragmatic hernia, which is a lethal birth defect characterized by lung hypoplasia and pulmonary hypertension, embryonic lungs showed a decrease in parasympathetic innervation and ASM peristalsis, as well as impaired branching (Pederiva et al., 2012; Rhodes et al., 2015).

Another type of pulmonary nerve tissue is part of the neuroendocrine system, and innervates the rare epithelial neuroendocrine (NE) cell type on the basal side by vagal nerve afferents (see references in Noguchi et al., 2015). NE cells often form clusters, called neuroepithelial bodies (NEBs), at airway branch bifurcation nodes (Kuo and Krasnow, 2015; Noguchi et al., 2015). These cells have been shown to be stem cells, and have been implicated as a source of small- and non-small cell lung cancers (Reynolds et al., 2000). NEBs have also been suggested to impact airway branching morphogenesis, possibly through paracrine signaling involving secreted neuropeptides and morphogenic factors (reviewed in Linnoila, 2006). An early paper on the role of NEBs in lung branching morphogenesis found that bombesin-like peptides, such as gastrin-releasing peptide (GRP), secreted by NEBs positively regulate branching morphogenesis in cultured E12 mouse lung buds (King et al., 1995). Furthermore, it was suggested that GRP expression might be regulated by fibronectin, which, as we have discussed, is highly expressed in branch clefts.

Very little exists in the literature concerning the mechanisms regulating pulmonary innervation and NEBs in the context of branching morphogenesis, while no research, to our knowledge, has specified a role for Fgf signaling in pulmonary nerve development. For example, it was found in one study that retinoic acid (RA) signaling was able to rescue deficient pulmonary innervation and ASM peristalsis in a model of congenital diaphragmatic hernia (Pederiva et al., 2012). RA signaling is a well-known regulator of branching morphogenesis, and acts upstream of Fgf10 during early lung development (reviewed in Fernandes-Silva et al., 2020), but whether it coordinates the rescue of innervation and peristalsis with Fgf signaling is unknown.

In another paper, it was found that developing ASM in the mouse produces brain-derived neurotrophic factor (Bdnf), which guides extending axons to the ASM to enable innervation (Radzikinas et al., 2011). These authors discovered that miR-206 acts to inhibit *Bdnf* expression post-transcriptionally in ASM, and that miR-206 is itself downregulated by Shh. Thus, as Shh secretion from airway epithelium increases, so too does the expression of BDNF in adjacent ASM along with increased innervation. Since it is well-established that Shh is downstream of Fgf signaling during branching morphogenesis, it would be interesting to study whether the Shh/miR-206/Bdnf signaling cascade is under Fgf control (Figure 7C).

Clearly, further work is needed to uncover the mechanisms regulating nerve and neuroendocrine development during lung organogenesis. Recent work from Xin Sun and colleagues underscores this point (e.g., see Branchfield et al., 2016; Sui et al., 2018; Garg et al., 2019). For example, pulmonary NE cells respond to external stimuli, such as allergens, and coordinate the response of the lung by communicating with the nervous system, by releasing neuropeptides and neurotransmitters, and by interacting with immune responses. NE cells are also involved in repair after injury, and have been implicated in numerous lung diseases, and are therefore potential targets of therapeutic intervention (see references in Garg et al., 2019).

## Theoretical Considerations

A major theme of this review is that lung branching morphogenesis is controlled by a host of factors at multiple biological and physical scales. This reflects the basic fact that biological organisms and structures are complex systems; the influence of a single constituent part reverberates through, and more or less impacts, the entirety of the system. Indeed, the orchestration of morphogenesis can appear irreducibly complex. As such, computationally modeling biological systems in general, and branching morphogenesis in particular, has proved challenging. This is evidenced by the number of different models of lung branching morphogenesis which have been proposed (for reviews, see Iber and Menshykau, 2013; Miura, 2015; Varner and Nelson, 2017; Lang et al., 2018). Here, we briefly discuss two compatible classes of models which continue to garner interest and support: fractal-based geometric models and ligand-receptor based Turing models.

### Fractal Geometry

**Figure 8:**
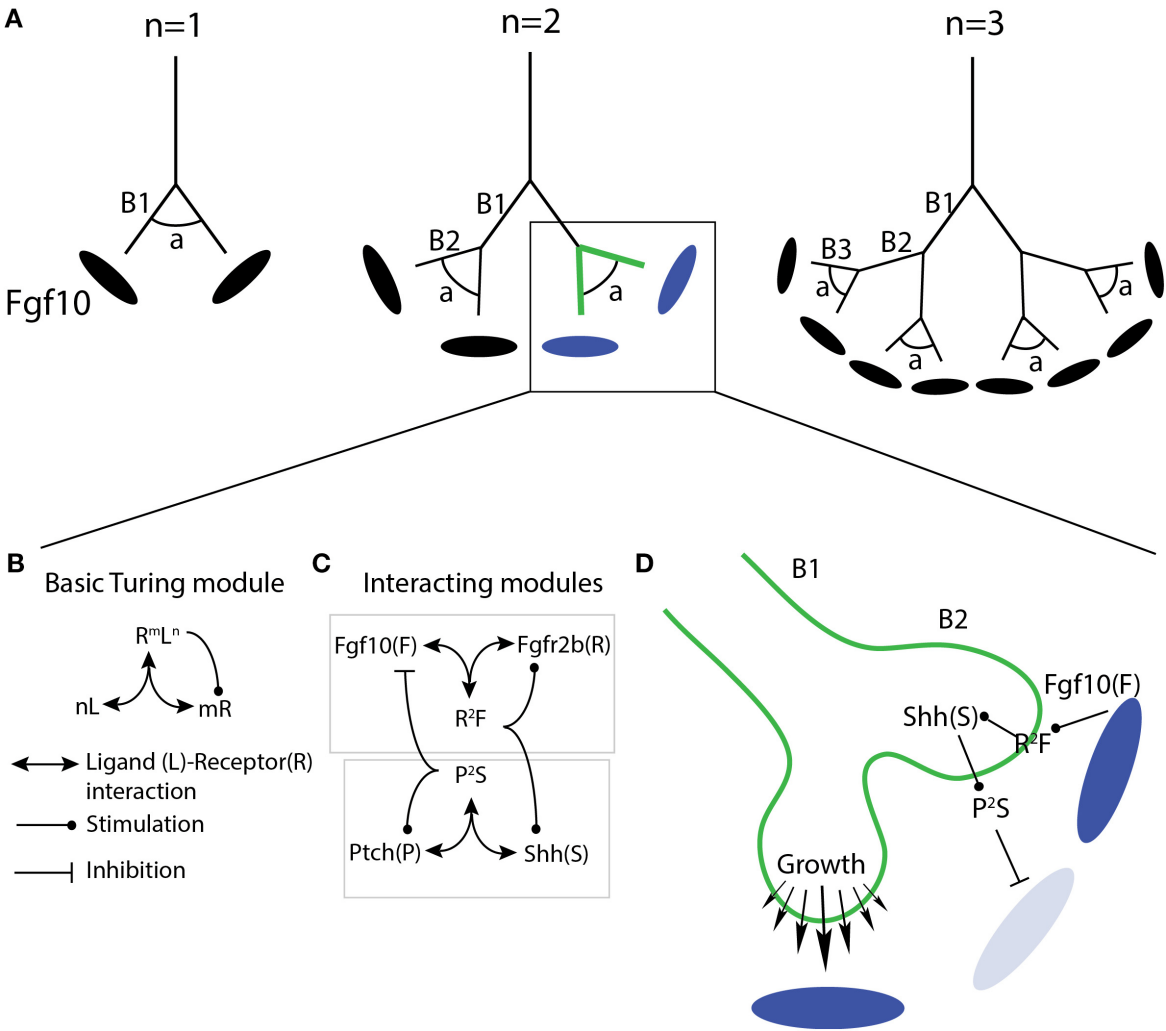
Model of branching morphogenesis based on fractal geometry controlled by ligand-receptor based Turing mechanisms. **(A)** Three generations (*n*) of branch tip bifurcation following fractal rules wherein the angle of bifurcation (a) is consistent among generations, and the length of each successive branch **(B)** is shorter than the previous branch. Each tip grows toward a domain of Fgf10 expression. **(B–D)** A single branching event (box in ‘A’) is modeled using a ligand-receptor based Turing mechanism. **(B)** The basic Turing module contains n copies of a ligand (L) interacting with m copies of a receptor (R). The combined ligand-receptor interaction stimulates increased expression of the receptor. **(C)** Two Turing modules, Fgf10 and Shh, which interact in a negative feedback to control airway branching. Fgf10 combines with two copies of Fgfr2b, which stimulates expression of Fgfr2b and of Shh. Shh combines with two copies of Ptch, which stimulates expression of Ptch and represses expression of Fgf10. **(D)** Two daughter branches (B2) bifurcate from the parent branch (B1) as a result of the interacting ligand-receptor based Turing modules containing Fgf10 and Shh. Simulations using these two Turing modules predict the observed mesenchymal patterning of Fgf10 expression (blue pools), as well as predicting the observed growth fields (different sized arrows) of cultured embryonic lung explants. See text for further details.

When the geometric properties of a branched network are mathematically related, it is, in principle, possible to model those properties. For instance, early work on human lung architecture reported that the average airway diameter (d) of the zth generation of dichotomous branching, d(z), can be calculated using the scaling equation d(z) = (d_0_)(2^−z/3^), where d_0_ is the average diameter of the zeroth generation (Weibel and Gomez, 1962). This equation captures a fundamental principle of airway branching morphogenesis, namely, that distal branches are not only smaller than more proximal parent branches, but also that branch geometries are related mathematically (Figure 8A).

The geometric scaling property of airway branches reflects the self-similarity between branching generations, which motivated one of the earliest attempts to model airway architecture using fractal rules (Mandelbrot and Wheeler, 1983). In fractal geometry, the geometry of a structure at one scale reconstitutes the geometry of a parent or daughter structure at a higher or lower scale, respectively. Daughter airway branch lengths, for example, are related to the parent branch length *via* the fractal dimension, which relates the similarity in length between daughter and parent branches. Interestingly, recent calculations of four commonly used laboratory mouse strains found that the fractal dimensions of conducting airways differed between strains (Glenny et al., 2020). This suggests not only that lung geometry is under genetic control, but also that care must be taken when comparing lung morphometry between different mouse strains.

Initial computer simulations of airway branching using fractal rules were able to produce branching structures, but failed to mirror the actual architecture of lung airways. This failure was in part due to the absence of any boundaries to fractal growth. However, once external boundaries matching the pleural cavity were imposed on the simulations, the fractal trees quantitatively approached those seen in nature (reviewed in Varner and Nelson, 2017).

A powerful feature of fractal geometries is that they can be computationally modeled using a small set of recursive rules (Glenny, 2011; Iber and Menshykau, 2013). Given that lung morphology is stereotyped and apparently built through a series of recursive instructions (Metzger et al., 2008), it is unsurprising that airway architecture shows fractal properties. However, no fractal-based simulation has yet to entirely recapitulate a natural airway tree (Varner and Nelson, 2017). This is likely a consequence of the intrinsic stochasticity inherent to any complex biological system, and to the fact that branching morphogenesis *is not only* determined by hard-wired genetic and molecular properties. Regardless, fractal-based simulations do point to a set of simple rules employed during branching morphogenesis. Recent research in computational and experimental biology have begun to uncover the biological mechanisms underlying these rules, and one class of models in particular is emerging as a strong candidate for a general theoretical explanation of branching morphogenesis: ligand-receptor based Turing models.

### Ligand-Receptor Based Turing Models

Turing models are named after the great Alan Turing, who devoted considerable effort studying and modeling the chemical basis of morphogenesis (Turing, 1952). In this work, Turing proposed a means by which patterned structures could emerge by the cooperative interaction of two or more substances, termed morphogens, from an initially homogenous, non-patterned state. In these models, self-organized patterned structures emerge from diffusion-driven instabilities in the otherwise homogenous initial conditions. Turing proposed that these instabilities can begin from inherent randomness in the system. Once initiated, however, the instability is self-reinforcing.

Turing patterns typically depend on at least two interacting factors that diffuse at different rates, and which results in the upregulation of one of the factors. This is generally the case for ligand-receptor systems, including, it has been suggested, Fgf10-Fgfr2b (reviewed in Iber and Menshykau, 2013; Lang et al., 2018). For example, a ligand-receptor Turing model was proposed by Menshykau et al. (2012) to account for branch mode selection during early lung branching morphogenesis. In this paper, the authors recapitulated observed patterns of branching both in wildtype and mutant mice by modeling published interactions between Fgf10, Shh, and the Shh receptor patched (Ptch). Varying the parameters controlled by this Fgf10-Shh-Ptch regulatory axis, the authors were able to simulate the different branching modes observed in nature. Furthermore, in two later papers from the same lab, the authors not only looked at how Turing mechanisms could, in principle, evolve in nature (Kurics et al., 2014), but they also applied their simulations to actual 2D and 3D geometric data-sets for the first time (Menshykau et al., 2014).

In the first of these papers (Kurics et al., 2014), the authors addressed how Turing patterns could arise and evolve *via* natural selection. In the most basic ligand-receptor Turing model (Figure 8B), simulations predict that only a small parameter space (the space of permitted values) exists for such a system to operate. In other words, given such a small range of diversity, it is hard to explain how such systems could evolve. However, when simulations are programmed to better account for actual natural systems, the parameter space drastically enlarges. For example, combining the negative feedback between two interacting Turing systems during branching morphogenesis—Fgf10/Fgfr2b and Shh/Ptch—the parameter space became huge (Figure 8C). In addition, the authors showed that the restriction of receptors to single cells, which is often the case in natural systems, further enlarges the parameter space.

In the second paper mentioned above (Menshykau et al., 2014), the authors applied the principles from the first paper, such as negative feedback between Fgf and Shh signaling, as well as the actual geometrically-restricted expression of ligands and receptors in embryonic mouse lungs. Here, 3D geometric datasets of embryonic mouse lungs, as well as 2D time-lapse imaging of lung explant cultures, were used to obtain physiological geometries and growth displacement fields. It was found that only a ligand-receptor based Turing mechanism, in cooperation with the geometrically-restricted patterning of receptor and ligand expressions, was able to predict the actual displacement fields and direction of growth in the lung samples (see Figure 8D).

This finding also explains the observed stereotypy of lung branching morphogenesis in the following way: in complex domains (such as those found in biological systems), Turing mechanisms alone yield different patterns for the same parameter set if initial conditions differ slightly because of noise, but the observed stereotypy in lung branching would be lost. However, the separation of ligands and receptors into distinct domains results in a geometry effect that pre-patterns the domain, which adds robustness to the system, enabling, when combined with the Turing mechanism, stereotypic branching. When ligands and receptors are co-expressed in the epithelium, it would be expected to result in a wide range of branching patterns, which is indeed observed in the kidney when GDNF is co-expressed with its receptor RET in the epithelium (Shakya et al., 2005). Therefore, the combination of the Turing mechanism with a geometry effect due to the separation of ligands and receptors into different domains is not only critical to obtain branching morphogenesis, but in a stereotypic fashion.

This work on Turing mechanisms in cooperation with geometric patterning of ligands and receptors has formed the foundation of a potentially powerful predictive model of lung branching morphogenesis (Blanc et al., 2012; Clément et al., 2012; Menshykau et al., 2014). These mechanisms are robust, and are not only able to overcome initial noisy conditions of a biological system, but can be generalized across systems. As such, for example, researchers should consider ligand-receptor based Turing explanations to account for the branching observed even when Fgf10 is ubiquitously expressed *in vivo* (i.e., Volckaert et al., 2013), or when denuded epithelium is cultured *in vitro* (see discussion and references in Varner and Nelson, 2017; Lang et al., 2018). As computational simulations illustrate, Turing mechanisms are dependent on choice of parameter values and signaling interactions. The exact values and interactions which exist in natural systems will have to be determined. Yet, these issues should be amenable to experimental testing, and as such, reveal an exciting avenue of research bridging theoretical and experimental biology.

## Summary and Conclusions

In this paper, we attempted to comprehensively review the regulation of airway branching morphogenesis in the context of Fgfr2b signaling in the mouse. Regarding the airway epithelium specifically, we focused on the three main stages of branching: bud initiation, branch elongation, and tip bifurcation. We combined research on different biological and physical scales, from the effects of Fgfr2b signaling on single epithelial cell responses (e.g., apical constriction), to mechanical stretching of entire epithelial tissue and intraluminal fluid pressure dynamics. We then discussed more indirect regulation of airway branching in the form of ECM remodeling, as well as the role of the pulmonary vascular and nerve networks. To round out the review, we touched upon exciting work in theoretical biology which uses computational and mathematical modeling, and in which Fgf10 signaling has proven to be a powerful component of these models. This “holistic” approach was intended to better capture the regulatory intricacies of airway branching.

Where should research efforts go from here? In terms of Fgfr2b signaling, ongoing work in each of the foci of branching morphogenesis covered in this review is expected to continue in animal and human models. For example, recent *in vitro* studies on early human lung branching morphogenesis have revealed apparent discrepancies in the role of Fgf10/Fgfr2b signaling between mice and humans (Danopoulos et al., 2019a,b). In this work, recombinant FGF10 added to embryonic human lung tissue explants resulted in hypoplastic and cystic branches, as opposed to the increased branching seen in mouse models. Whether this finding indicates a fundamental causal difference in Fgf10/Fgfr2b signaling between mice and humans is still a matter to be investigated. Perhaps, as a more recent study suggests, the apparent discrepancies observed to date might simply be due to an improper comparison of mouse and human embryonic stages (Taghizadeh et al., 2020).

Throughout this review, we have highlighted holes in our understanding which are in need of further investigation. For instance, is Fgfr2b signaling directly or indirectly involved in apical constriction during bud initiation, or in spindle orientation during branch elongation? Does Fgf10 directly regulate Tenascin-C expression during the mesenchymal remodeling involved in branching? And to what degree does Fgfr2b signaling inform the development of the pulmonary nerve network? Emerging research using embryonic lung-on-a-chip technology may be able to tackle these questions (Shrestha et al., 2020).

More generally, large gaps in our understanding of specific aspects of branching morphogenesis still exist. As already mentioned, for example, much is to be determined about the development and role of NE cells and bodies, as well as the remodeling of the ECM and mesenchyme. Furthermore, there is much to be learned using disease and injury models, and also from research on other branching organs which possibly share some common regulatory mechanisms to the lung. A good example of this can be found in studies on resident macrophages found in the mammary gland, where these cells play an active role during branching (Van Nguyen and Pollard, 2002; Gurusamy et al., 2014; Brady et al., 2017; Wilson et al., 2020). Whether resident macrophages play a similar instructive role during lung branching is yet to be determined. It is known, however, that macrophages are involved during lung inflammation and resulting branching impairments. For example, bronchopulmonary dysplasia (BPD) studies in mice have found that the inflammation characteristic of this disease disrupts airway branching morphogenesis. The inflammation response has been shown to be mediated by resident macrophages (Blackwell et al., 2011), leading to the activation of nuclear factor-kappaB (NF-kB) signaling, which directly impacts Fgf10 expression in the mesenchyme (Muraoka et al., 2000; Benjamin et al., 2010; Carver et al., 2013). Other studies on inflammation have also demonstrated a direct link between the immune response, Fgf10 signaling, and branching defects (Benjamin et al., 2007). Could it be that these immune cells also play an instructive role during normal lung development? The data that does exist in the literature, though sparse, seems to indicate they do. For example, research suggests that macrophages appear in the lung as early as E10 in the mesenchyme surrounding growing airway buds, and that as pseudoglandular development progresses, abundant levels of macrophages are located at branching points (Jones and Ricardo, 2013; Jones et al., 2013). Unfortunately, this research focused mostly on the role of macrophages in alveolarization, and yet, the authors note that in other organs, macrophages regulate morphogenesis through growth factor release, phagocytosis, and tissue patterning. During digit formation, for example, macrophages are involved in the clearance of interdigital webbing. In models where this function is perturbed, negative impacts are seen on the lungs, brain, and eye (Jones and Ricardo, 2013).

Finally, we would like to conclude with a word on the practical power of theoretical models of branching morphogenesis to potentially resolve conceptual issues. In the lung field, it was originally thought that localized mesenchymal Fgf10 expression was required for proper airway branching morphogenesis (e.g., Bellusci et al., 1997), but more recent evidence is commonly cited to suggest otherwise (recall Volckaert et al., 2013). The jury is still out on this issue, perhaps in part because the question has been improperly conceptualized. The ubiquitous mesenchymal expression of Fgf10 using an *Fgf10* null mice model, as was done by Volckaert et al. (2013), does not actually provide evidence one way or another concerning the need for localized Fgf10 expression. The major issue with this approach is the fact that Fgf10 was expressed ubiquitously, *including the regions where it is normally localized*; it is therefore not possible to determine, using this experimental model, whether those localized regions of signaling are truly dispensable for proper branch patterning.

Theoretical models can help resolve these issues. For example, using Turing concepts, what might be important in the context of Fgf10 signaling are the Turing patterns which arise, and which lead to branching pattern formation. It is the Turing pattern which might be localized, and not necessarily the Fgf10 expression (although, since evolution favors optimization of resources, it would make economic sense to focus Fgf10 expression where it is needed). Indeed, Turing patterns can emerge from initially noisy conditions, especially when multiple interacting Turing modules provide feedback, as is the case during lung branching (recall Kurics et al., 2014; Lang et al., 2018). Perhaps behind the noise of ubiquitous Fgf10 expression is a pattern nonetheless, a Turing pattern, that quickly becomes reinforced due to the feedback modules present on the epithelial surface to which Fgf10 signals. With these concepts in mind, researchers can begin designing experiments accounting for these interactions, which will better reflect the actual mechanisms underlying branching.

In conclusion, we hope to have demonstrated the multiple ways in which Fgfr2b signaling regulates the various aspects of airway branching morphogenesis. We also have attempted to bridge the different fields of research on branching morphogenesis to offer a more comprehensive understanding of this complex biological phenomenon. As research progresses, perhaps this big picture view can help clarify concepts and promote new ideas.

## Author Contributions

SB and MJ: writing, figure making, and editing. LC: figure making. All authors contributed to the article and approved the submitted version.

## Conflict of Interest

The authors declare that the research was conducted in the absence of any commercial or financial relationships that could be construed as a potential conflict of interest.
